# Supplementary material for: New approaches to meta-analyze differences in skewness, kurtosis, and correlation
Source: PLoS Biol. 2026 Feb 13;24(2):e3003653. doi: 10.1371/journal.pbio.3003653 (PMC12928594; doi:10.1371/journal.pbio.3003653)
Supplement: S1 File — (HTML) [file pbio.3003653.s001.html]

ONLINE SUPPLEMENT - Beyond sex differences in the mean: new approaches to meta-analyse differences in skewness, kurtosis, and correlation


## **Table of Contents**

- 1 Update
- 2 Introduction
- 3 Content
- 4 Prerequisites
  - 4.1 Loading packages
  - 4.2 Custom functions
- 5 Simulations
  - 5.1 Extended simulation details
  - 5.2 Extended simulation results
  - 5.3 Summary
- 6 Equations and custom functions to calculate effect sizes
  - 6.1 Skewness
  - 6.2 Kurtosis
  - 6.3 Zr
- 7 Data loading and preparation
- 8 Meta-analytical models
  - 8.1 Table A. Single variable effect sizes
  - 8.2 Table B. Correlational effect sizes
  - 8.3 Table C. Heterogeneity estimates
  - 8.4 Meta-analysis validation
- 9 Visualisations
  - 9.1 Scenarios explored for differences in skewness
  - 9.2 Scenarios explored for differences in kurtosis
  - 9.3 MA validation - visualisations
    - 9.3.1 Fig U
    - 9.3.2 Fig V
    - 9.3.3 Fig W
    - 9.3.4 Fig X
- 10 Software and package versions
- 11 Reference

# ONLINE SUPPLEMENT - Beyond sex differences in the mean: new approaches to meta-analyse differences in skewness, kurtosis, and correlation

 Code

- Show All Code
- Hide All Code
- ---
- View Source

Author

**Pietro Pollo, Szymon M. Drobniak, Hamed Haselimashhadi, Malgorzata Lagisz, Ayumi Mizuno, Laura A. B. Wilson, Daniel W. A. Noble, Shinichi Nakagawa**

Published

February 3, 2026

# 1 Update

We will update this tutorial when necessary. Readers can access the latest version in our GitHub repository.

If you have any questions, errors or bug reports, please contact Pietro Pollo (pietro\_pollo@hotmail.com) or Shinichi Nakagawa (snakagaw@ualberta.ca).

# 2 Introduction

This online material is a supplement to our paper “Beyond sex differences in mean: meta-analysis of differences in skewness, kurtosis, and correlation”. You will see how to calculate the new effect size statistics we have proposed and how to use them in a meta-analytical model using the `metafor` package in `R`.

# 3 Content

In this online material, we provide details on the simulations we ran to evaluate the effectiveness of our proposed effect size statistics (and their associated sampling error). We also show how to (1) calculate our newly proposed effect sizes (\(\Delta sk\), \(\Delta ku\), \(\Delta Zr\)) and (2) exemplify their use with data from the International Mouse Phenotyping Consortium.

# 4 Prerequisites

## 4.1 Loading packages

Our tutorial uses `R` statistical software and existing `R` packages, which you will first need to download and install.

If the packages are archived in CRAN, use `install.packages()` to install them. For example, to install the `metafor` , you can execute `install.packages("metafor")` in the console (bottom left pane of `R Studio`).

Version information of each package is listed at the end of this tutorial.

Code

```
if (!require("pacman")) {install.packages("pacman")}
pacman::p_load(corrr,
               DT,
               ggdist,
               ggtext,
               here,
               janitor,
               latex2exp,
               magick,
               metafor,
               orchaRd,
               pander,
               patchwork,
               tidyverse)

options(DT.options = list(rownames = FALSE,
                          dom = "Blfrtip",
                          scrollX = TRUE,
                          pageLength = 5,
                          columnDefs = list(list(targets = '_all', 
                                                 className = 'dt-center')),
                          buttons = c('copy', 
                                      'csv', 
                                      'excel', 
                                      'pdf')))

source("layout.R")
```

## 4.2 Custom functions

We also provide some additional helper functions to calculate effect sizes, process data, and visualise our results. The most straightforward way to use these custom functions is to run the code chunk below. Alternatively, paste the code into the console and hit `Enter` to have `R` ‘learn’ these custom functions.

If you want to use these custom functions in your own data, you will need to change the variable names according to your own data (check out the `R` code and you will see what we mean).

Code

```
# calculate established effect sizes ----
calc.effect <- function(data = raw_data, 
                        m) { # calculates other already established effect size statistics 
  escalc(measure = m,
         m1i = data$mean_male,
         m2i = data$mean_female,
         sd1i = data$sd_male,
         sd2i = data$sd_female,
         n1i = data$n_male,
         n2i = data$n_female,
         var.names = c(paste0(m,
                              "_est"),
                       paste0(m,
                              "_var")))
}

# processing functions ----
process.ind_effects <- function(chosen_trait = "fat_mass",
                                measure = "KU_delta",
                                output = "results") {
  ind_effects <-
    df_meta_analysed %>% 
    ungroup() %>% 
    filter(trait_name == chosen_trait,
           phenotyping_center %in% c("CCP-IMG",
                                     "HMGU",
                                     "JAX",
                                     "MRC H",
                                     "TCP")) %>% 
    mutate(type = "individual",
           n0 = n_male * n_female / n_total) %>% 
    select(phenotyping_center,
           strain_fig,
           n = n_total,
           n0,
           est = all_of(paste0(measure, 
                               "_",
                               "est")),
           var = all_of(paste0(measure, 
                               "_",
                               "var")),
           lower = all_of(paste0(measure, 
                                 "_",
                                 "lower")),
           upper = all_of(paste0(measure, 
                                 "_",
                                 "upper"))) %>% 
    rowid_to_column("effect_size_id")
  
  if (measure == "KU_delta") {
    # getting n0
    vtilde <- 1/ind_effects$n0
    
    ind_effects$sampling_error_id <- 
      ind_effects$effect_size_id
    
    Vf <- diag(as.numeric(vtilde))
    levs <- levels(factor(ind_effects$sampling_error_id))
    rownames(Vf) <- levs
    colnames(Vf) <- levs
    
    model <- 
      rma.mv(data = ind_effects,
             yi = est,
             V = 0,
             random = list(~ 1|effect_size_id,
                           ~ 1|sampling_error_id,  # this is like sampling error bit which usually goes to V
                           ~ 1|phenotyping_center, 
                           ~ 1|strain_fig),
             method = "REML",
             test = "t",
             R = list(sampling_error_id = Vf),
             Rscale = FALSE,
             # new optimizer
             control = list(rel.tol = 1e-8))
    
  } else {
    model <- rma.mv(data = ind_effects,
                    yi = est,
                    V = var,
                    method = "REML",
                    test = "t",
                    random = list(~ 1|effect_size_id,
                                  ~ 1|phenotyping_center, 
                                  ~ 1|strain_fig))
  }
  
  if (output == "results") {
    df_model <- data.frame(est = model$beta[1],
                           var = model$se ^ 2,
                           lower = model$ci.lb,
                           upper = model$ci.ub,
                           phenotyping_center = "Mean",
                           strain_fig = "ES")
    
    ind_effects %>% 
      bind_rows(df_model) %>% 
      mutate(est_type = measure,
             trait_name = chosen_trait,
             centre_and_strain = factor(paste0(phenotyping_center,
                                               "\n",
                                               strain_fig),
                                        levels = rev(c("CCP-IMG\nNCrl",
                                                       "HMGU\nNCrl",
                                                       "HMGU\nNTac",
                                                       "JAX\nNJ",
                                                       "MRC H\nNTac",
                                                       "TCP\nNCrl",
                                                       "Mean\nES"))))
    
  } else if (output == "heterogeneity") {
    if (measure == "KU_delta") {
      sampling_variance <- 
        model$sigma2[2] * vtilde
      
      # from Higgins - https://doi.org/10.1002/sim.1186
      av_sv <- 
        sum(1 / sampling_variance) / 
        sum((1 / sampling_variance) ^ 2) * sum(1 / sampling_variance) ^ 2 / 
        (sum(1 / sampling_variance) * (length(sampling_variance) - 1))
      
      denominator <-
        av_sv + model$sigma2[1] + model$sigma2[3] + model$sigma2[4]
      
      tibble(trait_name_1 = chosen_trait,
             trait_name_2 = NA,
             est_type = measure) %>% 
        add_column(i2_total = (model$sigma2[1] + model$sigma2[3] + model$sigma2[4]) * 100 /
                     denominator,
                   i2_effect_size_id = model$sigma2[1] * 100 / denominator,
                   i2_phenotyping_center = model$sigma2[3] * 100 / denominator,
                   i2_strain_fig = model$sigma2[4] * 100 / denominator) %>% 
        clean_names()
    } else {
      tibble(trait_name_1 = chosen_trait,
             trait_name_2 = NA,
             est_type = measure) %>% 
        add_column(as_tibble(t(i2_ml(model)))) %>% 
        clean_names()
    }
  }
}

process.cor_effects <- 
  function(chosen_trait_1 = "fat_mass",
           chosen_trait_2 = "heart_weight",
           output = "results") {
    
    df_raw_cor <-
      df_raw %>% 
      filter(trait_name %in% c(chosen_trait_1,
                               chosen_trait_2), 
             phenotyping_center %in% c("CCP-IMG",
                                       "HMGU",
                                       "JAX",
                                       "MRC H",
                                       "TCP")) %>% 
      pivot_wider(id_cols = c(specimen_id,
                              strain_fig,
                              phenotyping_center,
                              sex),
                  names_from = trait_name) %>%
      clean_names() %>% 
      drop_na() %>% 
      group_by(strain_fig,
               phenotyping_center,
               sex) %>% 
      group_modify(~ correlate(.x)) %>% 
      drop_na(all_of(chosen_trait_2)) %>% 
      ungroup() %>%
      left_join(df_raw %>% 
                  filter(trait_name %in% c(chosen_trait_1,
                                           chosen_trait_2), 
                         phenotyping_center %in% c("CCP-IMG",
                                                   "HMGU",
                                                   "JAX",
                                                   "MRC H",
                                                   "TCP")) %>% 
                  pivot_wider(id_cols = c(specimen_id,
                                          strain_fig,
                                          phenotyping_center,
                                          sex),
                              names_from = trait_name) %>%
                  clean_names() %>% 
                  drop_na() %>% 
                  group_by(strain_fig,
                           phenotyping_center,
                           sex) %>% 
                  summarise(n = n())) %>% 
      select(- all_of(chosen_trait_1)) %>% 
      rename(r_est = chosen_trait_2) %>% 
      pivot_wider(id_cols = c(strain_fig,
                              phenotyping_center),
                  names_from = sex,
                  values_from = c(r_est,
                                  n))
    
    df_effects_cor <-
      df_raw_cor %>% 
      left_join(df_raw_cor %>% 
                  group_by(phenotyping_center,
                           strain_fig) %>% 
                  reframe(orchaRd::cor_diff(cor1 = na.omit(r_est_male),
                                            cor2 = na.omit(r_est_female),
                                            n1 = na.omit(n_male),
                                            n2 = na.omit(n_female)))) %>% 
      rename(delta_zr_est = zr_diff,
             delta_zr_var = var_zr_diff) %>% 
      mutate(delta_zr_upper = delta_zr_est + qt(0.975, 
                                                n_male + n_female - 2) * sqrt(delta_zr_var),
             delta_zr_lower = delta_zr_est - qt(0.975, 
                                                n_male + n_female - 2) * sqrt(delta_zr_var)) %>% 
      rowid_to_column("effect_size_id")
    
    model <-
      rma.mv(data = df_effects_cor,
             yi = delta_zr_est,
             V = delta_zr_var,
             test = "t",
             random = list(~ 1|effect_size_id,
                           ~ 1|phenotyping_center, 
                           ~ 1|strain_fig))
    
    if (output == "results") {
      df_model <- data.frame(delta_zr_est = model$beta[1],
                             delta_zr_lower = model$ci.lb,
                             delta_zr_upper = model$ci.ub,
                             phenotyping_center = "Mean",
                             strain_fig = "ES")
      
      df_effects_cor %>% 
        bind_rows(df_model) %>% 
        mutate(centre_and_strain = factor(paste0(phenotyping_center,
                                                 "\n",
                                                 strain_fig))) %>% 
        mutate(centre_and_strain = factor(centre_and_strain,
                                          levels = rev(c("CCP-IMG\nNCrl",
                                                         "HMGU\nNCrl",
                                                         "HMGU\nNTac",
                                                         "JAX\nNJ",
                                                         "MRC H\nNTac",
                                                         "TCP\nNCrl",
                                                         "Mean\nES"))))
    } else if (output == "heterogeneity") {
      tibble(trait_name_1 = chosen_trait_1,
             trait_name_2 = chosen_trait_2,
             est_type = "delta_zr") %>% 
        add_column(as_tibble(t(i2_ml(model)))) %>% 
        clean_names()
    }
  }

# visualisation functions ----
caterpillar.custom <- 
  function(chosen_trait = "fat_mass",
           measure = "KU_delta") {
    
    df <-
      process.ind_effects(chosen_trait = chosen_trait,
                          measure = measure)
    
    plot <-
      df %>% 
      ggplot(aes(y = centre_and_strain,
                 x = est,
                 xmax = upper,
                 xmin = lower,
                 shape = strain_fig,
                 col = phenotyping_center,
                 size = as.factor(phenotyping_center == "Mean"))) +
      geom_pointrange() +
      geom_vline(xintercept = 0,
                 linetype = "dotted") +
      annotate(geom = "label",
               # x = df$est,
               x = 0,
               y = as.numeric(df$centre_and_strain) - 0.28,
               label = paste0(round(df$est, 
                                    2),
                              " (",
                              round(df$lower, 
                                    2),
                              ", ",
                              round(df$upper, 
                                    2),
                              ")"),
               size = 2.4,
               label.size = 0,
               fill = "white",
               col = "black") +
      scale_shape_manual(values = c(18:15)) +
      scale_size_manual(values = c(0.5,
                                   0.7)) +
      theme_classic() +
      theme(legend.position = "none",
            axis.text.y = element_blank(),
            axis.title.y = element_blank(),
            plot.tag.position = c(0.15, 
                                  0.98))
    
    lab_x <- 
      case_when(measure == "ROM" ~ "lnRR",
                measure == "VR" ~ "lnVR",
                measure == "SK_delta" ~ "&Delta;*sk*",
                measure == "KU_delta" ~ "&Delta;*ku*")
    
    if (chosen_trait %in% c("fat_mass",
                            "heart_weight")) {
      lim_x <-
        case_when(measure == "ROM" ~ 0.5,
                  measure == "VR" ~ 1,
                  measure == "SK_delta" ~ 3,
                  measure == "KU_delta" ~ 45)
    } else {
      lim_x <-
        case_when(measure == "ROM" ~ 0.5,
                  measure == "VR" ~ 0.5,
                  measure == "SK_delta" ~ 1,
                  measure == "KU_delta" ~ 6)
    }
    
    plot +
      labs(x = lab_x) +
      scale_x_continuous(limits = c(- lim_x * 1.02, 
                                    lim_x * 1.02),
                         breaks = c(- lim_x, 
                                    0, 
                                    lim_x)) +
      theme(axis.title.x = ggtext::element_markdown(face = "italic"))
  }

ridgeline.custom <- function(chosen_trait = "fat_mass") {
  
  processed_data <-
    df_raw %>% 
    filter(trait_name == chosen_trait,
           phenotyping_center %in% c("CCP-IMG",
                                     "HMGU",
                                     "JAX",
                                     "MRC H",
                                     "TCP")) %>% 
    add_row(phenotyping_center = "Mean",
            strain_fig = "ES") %>% 
    mutate(centre_and_strain = factor(paste0(phenotyping_center,
                                             "\n",
                                             strain_fig))) %>% 
    mutate(centre_and_strain = factor(centre_and_strain,
                                      levels = rev(c("CCP-IMG\nNCrl",
                                                     "HMGU\nNCrl",
                                                     "HMGU\nNTac",
                                                     "JAX\nNJ",
                                                     "MRC H\nNTac",
                                                     "TCP\nNCrl",
                                                     "Mean\nES"))),
           value_s = scale(value)[, 1])
  
  sample_sizes <-
    processed_data %>% 
    count(centre_and_strain,
          sex) %>% 
    slice(-1) %>% 
    add_row(centre_and_strain = factor("Mean\nES"),
            processed_data %>% 
              filter(!is.na(sex)) %>% 
              count(sex)) %>% 
    pivot_wider(id_cols = centre_and_strain,
                names_from = sex,
                values_from = n) %>% 
    mutate(label = paste0("Nf = ",
                          female,
                          ", Nm = ",
                          male)) %>% 
    arrange(centre_and_strain)
  
  processed_data %>% 
    ggplot(aes(x = value_s,
               y = centre_and_strain,
               fill = sex,
               linetype = sex)) +
    stat_slab(scale = 0.7, 
              alpha = 0.4,
              linewidth = 0.6,
              col = "black") +
    scale_fill_manual(values = c("white",
                                 "black")) +
    scale_linetype_manual(values = c("solid",
                                     "dashed")) +
    labs(x = paste0(str_to_sentence(str_replace_all(chosen_trait,
                                                    "_",
                                                    " ")),
                    "\n(scaled)"),
         y = "Phenotyping centre and mice strain") +
    annotate(geom = "text",
             x = mean(range(processed_data$value_s,
                            na.rm = T)),
             y = as.numeric(sample_sizes$centre_and_strain) - 0.15,
             label = sample_sizes$label,
             size = 2.3) +
    theme_classic() +
    theme(legend.position = "none",
          axis.title.x = element_text(size = 12, 
                                      margin = margin(t = 0.2,
                                                      unit = "cm")),
          axis.title.y = element_text(size = 12, 
                                      margin = margin(r = 0.2,
                                                      unit = "cm")),
          axis.text.x = element_text(size = 10),
          axis.text.y = element_text(size = 10),
          plot.tag.position = c(0.53, 
                                0.98))
}

cor.caterpillar.custom <- 
  function(chosen_trait_1 = "fat_mass",
           chosen_trait_2 = "heart_weight") {
    
    df <-
      process.cor_effects(chosen_trait_1 = chosen_trait_1,
                          chosen_trait_2 = chosen_trait_2)
    
    df %>% 
      ggplot(aes(y = centre_and_strain,
                 x = delta_zr_est,
                 xmax = delta_zr_upper,
                 xmin = delta_zr_lower,
                 shape = strain_fig,
                 col = phenotyping_center,
                 size = as.factor(phenotyping_center == "Mean"))) +
      geom_pointrange() +
      geom_vline(xintercept = 0,
                 linetype = "dotted") +
      annotate(geom = "label",
               # x = df$delta_zr_est,
               x = 0,
               y = as.numeric(df$centre_and_strain) - 0.15,
               label = paste0(round(df$delta_zr_est, 
                                    2),
                              " (",
                              round(df$delta_zr_lower, 
                                    2),
                              ", ",
                              round(df$delta_zr_upper, 
                                    2),
                              ")"),
               size = 2.4,
               label.size = 0,
               fill = "white",
               col = "black") +
      labs(y = "Phenotyping centre and mice strain",
           x = "&Delta;*Zr*", 
           shape = "Strain") +
      scale_x_continuous(limits = c(-1, 
                                    1),
                         breaks = c(-1, 
                                    0, 
                                    1)) +
      scale_shape_manual(values = c(18:15)) +
      scale_size_manual(values = c(0.6,
                                   0.8)) +
      theme_classic() +
      theme(legend.position = "none",
            axis.title.x = ggtext::element_markdown(size = 12, 
                                                    margin = margin(t = 0.2,
                                                                    unit = "cm")),
            axis.title.y = element_text(size = 12,
                                        margin = margin(r = - 0.1,
                                                        unit = "cm")),
            axis.text.x = element_text(size = 10),
            axis.text.y = element_text(size = 10),
            plot.tag.position = c(0.3, 
                                  0.99))
  }

cor.plot.custom <- 
  function(chosen_trait_1 = "fat_mass",
           chosen_trait_2 = "heart_weight",
           chosen_lims = c(-3, 5)) {
    df_cor <-
      df_raw %>% 
      filter(trait_name %in% c(chosen_trait_1,
                               chosen_trait_2), 
             phenotyping_center %in% c("CCP-IMG",
                                       "HMGU",
                                       "JAX",
                                       "MRC H",
                                       "TCP")) %>% 
      pivot_wider(id_cols = c(specimen_id,
                              strain_fig,
                              phenotyping_center,
                              sex),
                  names_from = trait_name) %>%
      clean_names() %>% 
      drop_na() %>% 
      mutate(centre_and_strain = factor(paste0(phenotyping_center,
                                               strain_fig))) %>% 
      mutate(trait_1_s = scale(get(chosen_trait_1))[,1],
             trait_2_s = scale(get(chosen_trait_2))[,1])
    
    sample_sizes <-
      df_cor %>% 
      count(centre_and_strain,
            sex) %>% 
      pivot_wider(id_cols = centre_and_strain,
                  names_from = sex,
                  values_from = n) %>% 
      mutate(label = paste0("Nf = ",
                            female,
                            ", Nm = ",
                            male)) %>% 
      arrange(desc(centre_and_strain))
    
    plot_list <- list()
    
    for (i in 1:length(levels(df_cor$centre_and_strain))) {
      level_i <- sort(levels(df_cor$centre_and_strain))[i]
      
      plot <-
        df_cor %>% 
        filter(centre_and_strain == level_i) %>% 
        ggplot(aes(x = trait_1_s,
                   y = trait_2_s,
                   shape = sex,
                   linetype = sex)) +
        geom_point(alpha = 0.008) +
        geom_abline(intercept = 0,
                    slope = 1,
                    linewidth = 0.5,
                    linetype = "dotted") +
        geom_smooth(method = "lm",
                    se = F,
                    col = "black") +
        scale_shape_manual(values = c(3, 
                                      4)) +
        scale_linetype_manual(values = c("solid",
                                         "dashed")) +
        scale_x_continuous(limits = chosen_lims) +
        scale_y_continuous(limits = chosen_lims) +
        labs(x = paste0(str_to_sentence(str_replace_all(chosen_trait_1, 
                                                        "_", 
                                                        " ")),
                        "\n(scaled)"),
             y = paste0(str_to_sentence(str_replace_all(chosen_trait_2, 
                                                        "_", 
                                                        " ")),
                        " (scaled)")) +
        annotate(geom = "label",
                 x = mean(chosen_lims),
                 y = 4.5,
                 label = sample_sizes$label[i],
                 size = 2.4,
                 label.size = 0) +
        theme_classic() +
        theme(legend.position = "none",
              plot.tag.position = c(0.05, 
                                    0.91),
              axis.title.x = element_text(size = 12, 
                                          margin = margin(t = 0.2,
                                                          unit = "cm")),
              axis.title.y = element_text(size = 12, 
                                          margin = margin(r = 0.2,
                                                          unit = "cm")),
              axis.text.x = element_text(size = 10),
              axis.text.y = element_text(size = 10))
      
      
      if (i != 6) {
        plot <-
          plot +
          theme(axis.title.x = element_blank(),
                axis.text.x = element_blank(),
                axis.line.x = element_blank(),
                axis.ticks.x = element_blank())
      }
      
      plot_list[[i]] <- plot
    }
    
    return(plot_list)
  }
```

# 5 Simulations

## 5.1 Extended simulation details

We conducted Monte-Carlo simulations to evaluate bias and variance estimation for our new effect sizes \(\Delta sk\), \(\Delta ku\), and \(\Delta Zr\). For \(\Delta sk\) and \(\Delta ku\) we simulated independent samples for two groups from Pearson distributions with known moments using the *rpearson* function from the PearsonDS R package (vers. 1.3.2 [1]). We conducted two simulations: 1) first by changing skewness between groups that involved moderate departures from normality (group-specific skewness, \(sk \in \{-1, -0.5, 0, 0.5, 1\}\) with kurtosis fixed at 3) and 2) by holding skewness constant (\(sk\) = 0) while manipulating kurtosis, \(ku \in \{2.5, 3, 4, 5, 6\}\). In all cases, we simulated scenarios where: (i) the variance between each group was the same (\(\sigma^2\_{2}\) = \(\sigma^2\_{1}\) = 1) or different (\(2\sigma^2\_{2}\) versus \(\sigma^2\_{1}\)); (ii) the mean between the two groups was the same (\(\mu\_{2}\) = \(\mu\_{1}\) = 0) or different (\(\mu\_{2}\) = 5, \(\mu\_{1}\) = 0). For simplicity we assumed equal sample sizes between groups with sample size varying from \(n \in \{10, 20, \dots, 100, 150, 500\}\). We created all unique combinations of the above scenarios resulting in 1200 independent scenarios (when considering each of the 100 scenarios at each sample size, see examples in Section 9.1 and Section 9.2). We estimated \(\Delta sk\) and \(\Delta ku\) for each scenario using formulas for within-group sample skewness with small-sample correction (Eq. 1 in main manuscript) and excess kurtosis with small-sample correction (Eq. 3 in main manuscript) to estimate point estimates. To estimate associated sampling variance for \(\Delta sk\) and \(\Delta ku\) we used the analytical variance estimators derived here and an associated re-sampling (jackknife) approach to compute group sampling variances separately followed by pooling. Importantly, our simulations assume no correlation between groups.

For \(\Delta Zr\) simulations, we simulated two groups each containing two variables with known correlations within each group. For \(\Delta Zr\) we drew bivariate normal data with target within-group correlations \(r \in \{-0.8, -0.4, -0.2, 0, 0.2, 0.4, 0.6, 0.8\}\) using the *mvnorm* function in the MASS package of R (vers. 7.3.61, [2]). Marginals were standard normal and group sizes varied from \(n \in \{10, 20, \dots, 100, 150, 500\}\). Again, we created all unique combinations of scenarios resulting in 768 unique scenarios. We estimated \(\Delta Zr\) using Fisher’s Z transformation \(Zr\) and calculating \(\Delta Zr\) as the difference of \(Zr\) across groups (Eqs. 9–11 in main manuscript). Sampling variance for \(\Delta Zr\) was approximated used the standard analytic variance \(\frac{1}{n-3}\) per group (summed; Eq. 10 in main manuscript) and a jackknife approach. Again, we assumed no correlation between our groups.

Across all simulations we conducted 2,500 replicates of each scenario. Performance metrics were (a) bias of the point estimator (mean estimate minus truth) (Equation 1), (b) relative bias of the sampling-variance estimator (Equation 2), (c) coverage (95%) and (d) Monte-Carlo standard errors (MCSEs) (Equation 3).

\[
\text{Bias}(\hat{\theta}\_{mu}) = \frac{\sum\_{i=1}^{n\_{\text{sim}}} \hat{\theta}\_i}{n\_{\text{sim}}} - \theta
\tag{1}\]

where \(\theta\_{mu}\) is the true overall mean effect (i.e., the true \(\Delta sk\), \(\Delta ku\), and \(\Delta Zr\) between groups), and \(\hat{\theta}\_i\) is the effect estimate from simulation \(i\).

We calcuated coverage as the proportion of 95% confidence intervals (CIs) that contained the true effect size across all simulations for each scenario.

To understand bias in the sampling variance, we computed the relative bias for the effect measure as:

\[
\text{Relative Bias}(\hat{\theta}\_{SV}) = \left( \frac{\mathbb{E}[\hat{SV}] - \hat{\theta}^2}{\hat{\theta}^2}\right) \cdot 100\%
\tag{2}\]

where \(\hat{\theta}^2\) is the estimated sampling variance for the effect measure and \(\mathbb{E}[\hat{SV}]\) is the expected value of the sampling variance. Relative bias can be calculated using different combinations of estimates. For example, we can use \(\hat{\theta}^2\) as the demoninator from a point estimate taken from the small sample correction for \(\Delta sk\), \(\Delta ku\), or \(\Delta Zr\) or from the jackknife estimate. Alternatively, we can use the jackknife estimate for \(\mathbb{E}[\hat{SV}]\) across each simulation. We created each of the four combinations for our performance measures. For each of our performance measures we also computed the Monte Carlo Standard Errors (MCSE) of the estimated bias with

\[
\text{MCSE} = \sqrt{\frac{S^2\_{\hat{\theta}}}{n\_{\text{sim}}}}
\tag{3}\]

where \(S^2\_{\hat{\theta}}\) is the sample variance of the estimated effects across simulations and \(n\_{\text{sim}}\) is the number of simulations.

## 5.2 Extended simulation results

In all cases, we found the Monte Carlo Sampling Error (MCSEs) to be low for all our performance metrics (\(\Delta sk\): range of MCSEs, 0 to 0.01; \(\Delta ku\): range of MCSEs, 0 to 0.6; \(\Delta Zr\): range of MCSEs, 0 to 0.004).

#### 5.2.0.1 Evaluating \(\Delta sk\), \(\Delta ku\), and \(\Delta Zr\) point estimators

Across all simulations, \(\Delta sk\), \(\Delta ku\), and \(\Delta Zr\) point estimators exhibited small sample bias with less than 20-30 samples, except for \(\Delta ku\), which continued to show this bias through to n ~ 50-60, indicating effect sizes involving kurtosis are more challenging to estimate (see Fig 2). Regardless, small sample biases were moderate, and there was rarely a consistent over or under-estimation in point estimates across the scenarios evaluated (see Fig 2).

As expected, both the analytical and jackknife estimates for \(\Delta sk\) and \(\Delta ku\) had minimal bias when distributions were normally distributed (Skewness of both groups = 0; Analytical bias= 0.001 with SD = 0.009 & Jackknife = 0.002 with SD = 0.011; Kurtosis of both groups = 3; Analytical bias=-0.005 with SD = 0.025 & Jackknife = -0.006 with SD = 0.031), but bias in both seemed to change in complex ways that suggest challenges in estimating moments (see S2 Fig). This did not appear to be related to differences in group means or variances as bias patterns were similar across these conditions (see S3 Fig), but rather the sampling distributions being skewed (see “Coverage” below).

Importantly, bias-corrected jackknife estimates reduced the small-sample bias relative to analytical bias corrected-moment estimators (see S1 Fig; \(\Delta sk\) Mean Square Bias (MSB): Jackknife: 1.109, Analytical: 3.375; \(\Delta ku\) Mean Square Bias (MSB): Jackknife: 473.737, Analytical: 886.836; \(\Delta cor\) Mean Square Bias (MSB): Jackknife: 72.733, Analytical: 535.089).

#### 5.2.0.2 Evaluating sampling variance estimators for \(\Delta sk\), \(\Delta ku\), and \(\Delta Zr\)

In contrast to point estimators, the effectiveness of sampling variance estimators for \(\Delta sk\), \(\Delta ku\), and \(\Delta Zr\) varied. Analytical sampling variance formulas for \(\Delta sk\) and \(\Delta ku\) were consistently biased (see S4 Fig). Jackknife resampling, when combined with analytical point estimates (see S4 Fig D, E), improved performance. Under these conditions, estimators performed well when n > 50. In contrast, the performance of sampling variance estimators for \(\Delta Zr\) was best when using the analytical formulas for both the point estimator and it’s associated sampling variance (see S4 Fig I).

#### 5.2.0.3 95% Coverage

Coverage was generally close to nominal levels (95%) for \(\Delta sk\) and \(\Delta Zr\), but slighly poorer when sample sizes were small (< n = 20) (see S5 Fig). For \(\Delta Zr\), coverage was close to nominal levels across all sample sizes when using the analytical sampling variance estimator (see S5 Fig). However, coverage was poor for \(\Delta ku\) across many scenarios no matter what estimator was used (see S5 Fig). Unintuitively, coverage for \(\Delta ku\) was often worse as sample sizes were larger (see S5 Fig), driven by the fact that point estimates were more accurately estimated with large sample sizes, but the sampling distribution became highly skewed, impacting coverage (see S6 Fig). At small sample sizes \(\Delta ku\) was estimated poorly when true \(\Delta ku\) was high, leading to non-skewed distributions with good coverage. In contrast, large sample sizes improved point estimation of \(\Delta ku\) when true differences existed, but the sampling distribution became highly skewed leading to poor coverage (see S6 Fig). As a sensitivity analysis, we also evaluated coverage when using bootstrap resampling to estimate sampling variances for all effect sizes. Results were qualitatively similar.

## 5.3 Summary

In light of these simulation results, we suggest pairing the formula-based point estimators for skewness (Eq. 1 in main manuscript) and kurtosis (Eq. 3 in main manuscript) with jackknife standard errors for \(\Delta sk\) and \(\Delta ku\). For \(\Delta Zr\), the standard analytic variance is recommended (Eqns. 9-12 in main manuscript). This choice balances efficiency under normality with robustness to realistic deviations from it, and aligns with our broader guidance to avoid very small group sizes for these statistics. Given the challenges in estimating \(\Delta ku\), and the poor properties of its sampling variance, we recommend weighted meta-analytic models using sample size instead of sampling variance.

# 6 Equations and custom functions to calculate effect sizes

## 6.1 Skewness

Following Joanes & Gill (1998).

\[
sk = \frac{\frac{1}{n} \sum\_{i = 1}^{n}(x\_{i} - \bar{x}) ^ 3}{[\frac{1}{n} \sum\_{i = 1}^{n}(x - \bar{x}) ^ 2] ^ \frac{3}{2}}
\frac{\sqrt{n (n - 1)}}{n - 2}
\] \[
s^2\_{sk} = \frac{6n(n - 1)}{(n - 2)(n + 1)(n + 3)}
\]

\[
\Delta sk = sk\_{1} - sk\_{2}
\]

\[
s^2\_{\Delta sk} = s^2\_{sk\_1} + s^2\_{sk\_2} - 2 \rho\_{sk} s\_{sk\_1} s\_{sk\_2}
\]

## 6.2 Kurtosis

Following Joanes & Gill (1998).

\[
ku = \frac{n (n + 1) (n - 1)}{(n - 2)(n - 3)}
\frac{\sum\_{i = 1}^{n}(x\_{i} - \bar{x}) ^ 4}
{[\sum\_{i = 1}^{n}(x\_{i} - \bar{x}) ^ 2]^ 2} -
\frac{3(n - 1) ^ 2}{(n - 2)(n - 3)}
\] \[
s^2\_{ku} = \frac{24 n (n - 1) ^ 2}{(n - 3)(n - 2)(n + 3)(n + 5)}
\]

\[
\Delta ku = ku\_{1} - ku\_{2}
\]

\[
s^2\_{\Delta ku} = s^2\_{ku\_1} + s^2\_{ku\_2} - 2 \rho\_{ku} s\_{ku\_1} s\_{ku\_2}
\]

## 6.3 Zr

Following Hedges & Olkin (1985).

\[
Zr = \frac{ln(\frac{1 + r}{1 - r})}{2}
\]

\[
s^2\_{Zr} = \frac{1}{n - 3}
\] \[
\Delta Zr = Zr\_{1} - Zr\_{2}
\]

\[
s^2\_{\Delta Zr} = s^2\_{Zr\_1} + s^2\_{Zr\_2} -2 \rho\_{Zr} s\_{Zr\_1} s\_{Zr\_2}
\]

# 7 Data loading and preparation

We use data from the International Mouse Phenotyping Consortium (IMPC, version 18.0; [3]; http://www.mousephenotype.org/).

Code

```
# raw data ----
df_raw <- 
  read_csv("mice_data_sample.csv") %>% 
  # small adjustments to make plots more readable:
  mutate(phenotyping_center = 
           ifelse(phenotyping_center == "MRC Harwell",
                  "MRC H",
                  phenotyping_center),
         strain_fig = case_when(strain_accession_id == "MGI:2159965" ~ 
                                  "N",
                                strain_accession_id == "MGI:2683688" ~ 
                                  "NCrl",
                                strain_accession_id == "MGI:2164831" ~ 
                                  "NTac",
                                strain_accession_id == "MGI:3056279" ~ 
                                  "NJ",
                                strain_accession_id == "MGI:2160139" ~ 
                                  "NJcl"))

df_raw_wide <-
  df_raw %>% 
  select(- strain_accession_id) %>% 
  pivot_wider(id_cols = c(specimen_id,
                          trait_name,
                          phenotyping_center,
                          strain_fig),
              names_from = sex,
              values_from = value)

df_meta_analysed <- # takes 30s to a minute to run
  df_raw %>% 
  group_by(sex,
           trait_name,
           phenotyping_center,
           strain_fig) %>% 
  summarize(mean = mean(value,
                        na.rm = T),
            sd = sd(value,
                    na.rm = T),
            n = n()) %>% 
  pivot_wider(id_cols = c(trait_name,
                          phenotyping_center,
                          strain_fig),
              names_from = sex,
              values_from = c(mean:n)) %>% 
  bind_cols(calc.effect(., m = "ROM")) %>% # lnRR
  bind_cols(calc.effect(., m = "CVR")) %>% # lnCVR
  bind_cols(calc.effect(., m = "VR")) %>%  # lnVR
  left_join(df_raw_wide %>% 
              group_by(trait_name,
                       phenotyping_center,
                       strain_fig) %>% 
              reframe(orchaRd::moment_effects(x1 = na.omit(male),
                                              x2 = na.omit(female),
                                              type = "skew"))) %>% 
  left_join(df_raw_wide %>% 
              group_by(trait_name,
                       phenotyping_center,
                       strain_fig) %>% 
              reframe(orchaRd::moment_effects(x1 = na.omit(male),
                                              x2 = na.omit(female),
                                              type = "kurt"))) %>% 
  rename(SK_delta_est = d_skew,
         SK_delta_var = d_skew_v,
         KU_delta_est = d_kurt,
         KU_delta_var = d_kurt_v) %>% 
  mutate(n_total = n_female + n_male,
         prop_females = n_female / (n_female + n_male)) %>% 
  select(trait_name,
         phenotyping_center,
         strain_fig,
         n_total,
         n_male,
         n_female,
         prop_females,
         ROM_est,
         ROM_var,
         CVR_est,
         CVR_var,
         VR_est,
         VR_var,
         SK_delta_est,
         SK_delta_var,
         KU_delta_est,
         KU_delta_var) %>% 
  mutate(ROM_upper = ROM_est + qt(0.975, 
                                  n_total - 1) * sqrt(ROM_var),
         ROM_lower = ROM_est - qt(0.975, 
                                  n_total - 1) * sqrt(ROM_var),
         CVR_upper = CVR_est + qt(0.975, 
                                  n_total - 1) * sqrt(CVR_var),
         CVR_lower = CVR_est - qt(0.975, 
                                  n_total - 1) * sqrt(CVR_var),
         VR_upper = VR_est + qt(0.975, 
                                n_total - 1) * sqrt(VR_var),
         VR_lower = VR_est - qt(0.975, 
                                n_total - 1) * sqrt(VR_var),
         SK_delta_upper = SK_delta_est + qt(0.975, 
                                            n_total - 1) * sqrt(SK_delta_var),
         SK_delta_lower = SK_delta_est - qt(0.975, 
                                            n_total - 1) * sqrt(SK_delta_var),
         KU_delta_upper = KU_delta_est + qt(0.975, 
                                            n_total - 1) * sqrt(KU_delta_var),
         KU_delta_lower = KU_delta_est - qt(0.975, 
                                            n_total - 1) * sqrt(KU_delta_var))
```

# 8 Meta-analytical models

We then use the data from multiple phenotyping centres and mice strains to calculate average effect sizes (\(\Delta sk\), \(\Delta ku\), and \(\Delta Zr\)).

## 8.1 Table A. Single variable effect sizes

Code

```
map2_dfr(.x = rep(c("fat_mass",
                    "heart_weight",
                    "glucose",
                    "total_cholesterol"),
                  each = 4),
         .y = rep(c("ROM",
                    "VR",
                    "SK_delta",
                    "KU_delta"), 
                  4),
         .f = process.ind_effects) %>% 
  dplyr::select(- c(effect_size_id,
                    sampling_error_id,
                    n0)) %>% 
  mutate(est_type = case_when(est_type == "ROM" ~ "lnRR",
                              est_type == "VR" ~ "lnVR",
                              est_type == "SK_delta" ~ "delta_sk",
                              est_type == "KU_delta" ~ "delta_ku")) %>% 
  datatable(.,
            extensions = "Buttons",
            rownames = FALSE)
```

## 8.2 Table B. Correlational effect sizes

Code

```
map2_dfr(.x = c("fat_mass",
                "glucose"),
         .y = c("heart_weight",
                "total_cholesterol"),
         .f = process.cor_effects) %>% 
  mutate(relationship = rep(c("fat mass and heart weight",
                              "glucose and total cholesterol"),
                            each = 7)) %>% 
  dplyr::select(- effect_size_id) %>% 
  relocate(relationship) %>%  
  datatable(.,
            extensions = "Buttons",
            rownames = FALSE)
## Warning: Using an external vector in selections was deprecated in tidyselect 1.1.0.
## ℹ Please use `all_of()` or `any_of()` instead.
##   # Was:
##   data %>% select(chosen_trait_2)
## 
##   # Now:
##   data %>% select(all_of(chosen_trait_2))
## 
## See <https://tidyselect.r-lib.org/reference/faq-external-vector.html>.
```

## 8.3 Table C. Heterogeneity estimates

Code

```
map2_dfr(.x = rep(c("fat_mass",
                    "heart_weight",
                    "glucose",
                    "total_cholesterol"),
                  each = 4),
         .y = rep(c("ROM",
                    "VR",
                    "SK_delta",
                    "KU_delta"), 
                  4),
         .f = ~ process.ind_effects(chosen_trait = .x,
                                    measure = .y,
                                    output = "heterogeneity")) %>% 
  mutate(est_type = case_when(est_type == "ROM" ~ "lnRR",
                              est_type == "VR" ~ "lnVR",
                              est_type == "SK_delta" ~ "delta_sk",
                              est_type == "KU_delta" ~ "delta_ku")) %>% 
  bind_rows(map2_dfr(.x = c("fat_mass",
                            "glucose"),
                     .y = c("heart_weight",
                            "total_cholesterol"),
                     .f = ~ process.cor_effects(chosen_trait_1 = .x,
                                                chosen_trait_2 = .y,
                                                output = "heterogeneity"))) %>% 
  datatable(.,
            extensions = "Buttons",
            rownames = FALSE)
```

## 8.4 Meta-analysis validation

Statistical validation of meta-analytic models was performed to study statistical error and bias in meta-analytic estimates, the influence of varying levels of heterogeneity, the impact of meta-analytical sample size, and the agreement between a summary-level MA and an IPD-type meta-analysis of idividual-level data. Simulations were performed along the following lines: 1. Original IMPC data was subsampled to generate datasets of varying sizes (k = 5, 10, 20, 40, 60, 80 “studies” per meta-analysis; within-study sample size of 50 or 100 individuals). 2. For each subsampled dataset, known effect size magnitude was introduced by Box-Cox transforming the data within sexes to generate known differences in skewness, or by introducing outliers to generate known differences in kurtosis. We generated three scenarions of effect-size magnitudes: small (0.3), medium (0.5), and large (1.0) for both skewness and kurtosis. 3. Meta-analytical models were fitted to each simulated dataset to estimate effect sizes, using the estimators and sampling variances recommended from the simulation study (see package `orchaRd` for details). Each simulation scenario was repeated 200 times.

See summary plots of the validations below (Figs U, V, W, X).

# 9 Visualisations

## 9.1 Scenarios explored for differences in skewness

- Fig A
- Fig B
- Fig C
- Fig D
- Fig E
- Fig F
- Fig G
- Fig H
- Fig I
- Fig J

Figure 1

Figure 2

Figure 3

Figure 4

Figure 5

Figure 6

Figure 7

Figure 8

Figure 9

Figure 10

## 9.2 Scenarios explored for differences in kurtosis

- Fig K
- Fig L
- Fig M
- Fig N
- Fig O
- Fig P
- Fig Q
- Fig R
- Fig S
- Fig T

Figure 11

Figure 12

Figure 13

Figure 14

Figure 15

Figure 16

Figure 17

Figure 18

Figure 19

Figure 20

## 9.3 MA validation - visualisations

### 9.3.1 Fig U

Fig U. MA validation results for skewness and kurtosis differences for MA of varying size (horizontal axis) and within-study sample size of 50 or 100 (colour). Vertical axis shows the power to detect small (0.3), medium (0.5), and large (1.0) effect sizes for kurtosis (top panel) and skewness (bottom panel).

### 9.3.2 Fig V

Fig V. MA validation results for skewness and kurtosis differences for MA of varying size (horizontal axis) and within-study sample size of 50 or 100 (colour). Vertical axis shows the estimation bias for small (0.3), medium (0.5), and large (1.0) effect sizes for kurtosis (top panel) and skewness (bottom panel).

### 9.3.3 Fig W

Fig W. MA validation results for skewness and kurtosis (colour-coded) differences for MA of varying heterogeneity (horizontal axis). Top panel - estimation bias of main effect size; bottom panel - mean estimated between-study variance (on log scale). Within-study sample size was set to 100 and effect size magnitude to medium (0.5). Plots show one selected trait (glucose levels).

### 9.3.4 Fig X

Fig X. Comparison of summary-level meta-analysis and individual participant data (IPD)-type meta-analysis for skewness and kurtosis differences. Top panel - main effect size estimates from both types of MA for a number of traits (shape-coded), and both effect size metrics (skeweness/kurtosis differences, colour-coded); dashed line indicates the `y~x` line. Bottom-panel - Bland-Altman plot comparing both approches. Within-study sample size was set to 100 and effect size magnitude to medium (0.5).

# 10 Software and package versions

Code

```
sessionInfo() %>% 
  pander()
```

**R version 4.5.1 (2025-06-13 ucrt)**

**Platform:** x86\_64-w64-mingw32/x64

**locale:** *LC\_COLLATE=English\_Australia.utf8*, *LC\_CTYPE=English\_Australia.utf8*, *LC\_MONETARY=English\_Australia.utf8*, *LC\_NUMERIC=C* and *LC\_TIME=English\_Australia.utf8*

**attached base packages:** *stats*, *graphics*, *grDevices*, *utils*, *datasets*, *methods* and *base*

**other attached packages:** *lubridate(v.1.9.4)*, *forcats(v.1.0.0)*, *stringr(v.1.5.1)*, *dplyr(v.1.1.4)*, *purrr(v.1.1.0)*, *readr(v.2.1.5)*, *tidyr(v.1.3.1)*, *tibble(v.3.3.0)*, *ggplot2(v.4.0.1)*, *tidyverse(v.2.0.0)*, *patchwork(v.1.3.2)*, *pander(v.0.6.6)*, *orchaRd(v.2.1.3)*, *metafor(v.4.8-0)*, *numDeriv(v.2016.8-1.1)*, *metadat(v.1.4-0)*, *Matrix(v.1.7-3)*, *magick(v.2.9.0)*, *latex2exp(v.0.9.8)*, *janitor(v.2.2.1)*, *here(v.1.0.2)*, *ggtext(v.0.1.2)*, *ggdist(v.3.3.3)*, *DT(v.0.34.0)*, *corrr(v.0.4.5)* and *pacman(v.0.5.1)*

**loaded via a namespace (and not attached):** *gtable(v.0.3.6)*, *bslib(v.0.9.0)*, *xfun(v.0.56)*, *htmlwidgets(v.1.6.4)*, *lattice(v.0.22-7)*, *mathjaxr(v.2.0-0)*, *tzdb(v.0.5.0)*, *crosstalk(v.1.2.2)*, *vctrs(v.0.7.0)*, *tools(v.4.5.1)*, *generics(v.0.1.4)*, *parallel(v.4.5.1)*, *pkgconfig(v.2.0.3)*, *RColorBrewer(v.1.1-3)*, *S7(v.0.2.0)*, *distributional(v.0.6.0)*, *lifecycle(v.1.0.5)*, *compiler(v.4.5.1)*, *farver(v.2.1.2)*, *snakecase(v.0.11.1)*, *sass(v.0.4.10)*, *htmltools(v.0.5.9)*, *yaml(v.2.3.12)*, *jquerylib(v.0.1.4)*, *crayon(v.1.5.3)*, *pillar(v.1.11.1)*, *cachem(v.1.1.0)*, *nlme(v.3.1-168)*, *tidyselect(v.1.2.1)*, *digest(v.0.6.39)*, *stringi(v.1.8.7)*, *rprojroot(v.2.1.1)*, *fastmap(v.1.2.0)*, *grid(v.4.5.1)*, *cli(v.3.6.5)*, *magrittr(v.2.0.3)*, *withr(v.3.0.2)*, *scales(v.1.4.0)*, *bit64(v.4.6.0-1)*, *timechange(v.0.3.0)*, *rmarkdown(v.2.30)*, *bit(v.4.6.0)*, *otel(v.0.2.0)*, *png(v.0.1-8)*, *hms(v.1.1.4)*, *evaluate(v.1.0.5)*, *knitr(v.1.51)*, *viridisLite(v.0.4.2)*, *rlang(v.1.1.7)*, *gridtext(v.0.1.5)*, *Rcpp(v.1.1.0)*, *glue(v.1.8.0)*, *xml2(v.1.4.0)*, *rstudioapi(v.0.18.0)*, *vroom(v.1.6.5)*, *jsonlite(v.2.0.0)* and *R6(v.2.6.1)*

# 11 Reference

1. Becker M, Klößner S. PearsonDS: Pearson distribution system. 2025. Available: https://cran.r-project.org/package=PearsonDS
2. Venables WN, Ripley BD. Modern applied statistics with S. New York, NY: Springer New York; 2002. doi:10.1007/978-0-387-21706-2
3. Dickinson ME, Flenniken AM, Ji X, Teboul L, Wong MD, White JK, et al. High-throughput discovery of novel developmental phenotypes. Nature. 2016;537: 508–514. doi:10.1038/nature19356


##### Source Code

```
---
title: "ONLINE SUPPLEMENT - Beyond sex differences in the mean: new approaches to meta-analyse differences in skewness, kurtosis, and correlation"
author: "**Pietro Pollo, Szymon M. Drobniak, Hamed Haselimashhadi, Malgorzata Lagisz, Ayumi Mizuno, Laura A. B. Wilson, Daniel W. A. Noble, Shinichi Nakagawa**"
date: "`r Sys.Date()`"
format:
  html:
    toc: true
    toc-location: left
    toc-depth: 3
    toc-title: "**Table of Contents**"
    output-file: "index.html"
    theme: simplex
    embed-resources: true
    code-fold: show
    code-tools: true
    number-sections: true
    bibliography: ref.bib
    fontsize: "12"
    max-width: "10"
    code-overflow: wrap
    page-layout: full
    fig-numbering: false
editor_options: 
  chunk_output_type: console
---

```{r setup}
#| include: false

knitr::opts_chunk$set(
  collapse = TRUE,
  message = FALSE,
  warnings = FALSE,
  echo = TRUE#,
  #comment = "#>"
)
```

# Update

We will update this tutorial when necessary. Readers can access the latest version in our [GitHub repository](https://github.com/pietropollo/new_effect_size_statistics).

If you have any questions, errors or bug reports, please contact Pietro Pollo (pietro_pollo\@hotmail.com) or Shinichi Nakagawa (snakagaw\@ualberta.ca).

# Introduction

This online material is a supplement to our paper "Beyond sex differences in mean: meta-analysis of differences in skewness, kurtosis, and correlation". You will see how to calculate the new effect size statistics we have proposed and how to use them in a meta-analytical model using the `metafor` package in `R`.

# Content

In this online material, we provide details on the simulations we ran to evaluate the effectiveness of our proposed effect size statistics (and their associated sampling error). We also show how to (1) calculate our newly proposed effect sizes ($\Delta sk$, $\Delta ku$, $\Delta Zr$) and (2) exemplify their use with data from the International Mouse Phenotyping Consortium.

# Prerequisites

## Loading packages

Our tutorial uses `R` statistical software and existing `R` packages, which you will first need to download and install.

If the packages are archived in CRAN, use `install.packages()` to install them.
For example, to install the `metafor` , you can execute `install.packages("metafor")` in the console (bottom left pane of `R Studio`).

Version information of each package is listed at the end of this tutorial.

```{r packages}
if (!require("pacman")) {install.packages("pacman")}
pacman::p_load(corrr,
               DT,
               ggdist,
               ggtext,
               here,
               janitor,
               latex2exp,
               magick,
               metafor,
               orchaRd,
               pander,
               patchwork,
               tidyverse)

options(DT.options = list(rownames = FALSE,
                          dom = "Blfrtip",
                          scrollX = TRUE,
                          pageLength = 5,
                          columnDefs = list(list(targets = '_all', 
                                                 className = 'dt-center')),
                          buttons = c('copy', 
                                      'csv', 
                                      'excel', 
                                      'pdf')))

source("layout.R")
```

## Custom functions

We also provide some additional helper functions to calculate effect sizes, process data, and visualise our results.
The most straightforward way to use these custom functions is to run the code chunk below.
Alternatively, paste the code into the console and hit `Enter` to have `R` ‘learn’ these custom functions.

If you want to use these custom functions in your own data, you will need to change the variable names according to your own data (check out the `R` code and you will see what we mean).

```{r}
#| code-fold: true

# calculate established effect sizes ----
calc.effect <- function(data = raw_data, 
                        m) { # calculates other already established effect size statistics 
  escalc(measure = m,
         m1i = data$mean_male,
         m2i = data$mean_female,
         sd1i = data$sd_male,
         sd2i = data$sd_female,
         n1i = data$n_male,
         n2i = data$n_female,
         var.names = c(paste0(m,
                              "_est"),
                       paste0(m,
                              "_var")))
}

# processing functions ----
process.ind_effects <- function(chosen_trait = "fat_mass",
                                measure = "KU_delta",
                                output = "results") {
  ind_effects <-
    df_meta_analysed %>% 
    ungroup() %>% 
    filter(trait_name == chosen_trait,
           phenotyping_center %in% c("CCP-IMG",
                                     "HMGU",
                                     "JAX",
                                     "MRC H",
                                     "TCP")) %>% 
    mutate(type = "individual",
           n0 = n_male * n_female / n_total) %>% 
    select(phenotyping_center,
           strain_fig,
           n = n_total,
           n0,
           est = all_of(paste0(measure, 
                               "_",
                               "est")),
           var = all_of(paste0(measure, 
                               "_",
                               "var")),
           lower = all_of(paste0(measure, 
                                 "_",
                                 "lower")),
           upper = all_of(paste0(measure, 
                                 "_",
                                 "upper"))) %>% 
    rowid_to_column("effect_size_id")
  
  if (measure == "KU_delta") {
    # getting n0
    vtilde <- 1/ind_effects$n0
    
    ind_effects$sampling_error_id <- 
      ind_effects$effect_size_id
    
    Vf <- diag(as.numeric(vtilde))
    levs <- levels(factor(ind_effects$sampling_error_id))
    rownames(Vf) <- levs
    colnames(Vf) <- levs
    
    model <- 
      rma.mv(data = ind_effects,
             yi = est,
             V = 0,
             random = list(~ 1|effect_size_id,
                           ~ 1|sampling_error_id,  # this is like sampling error bit which usually goes to V
                           ~ 1|phenotyping_center, 
                           ~ 1|strain_fig),
             method = "REML",
             test = "t",
             R = list(sampling_error_id = Vf),
             Rscale = FALSE,
             # new optimizer
             control = list(rel.tol = 1e-8))
    
  } else {
    model <- rma.mv(data = ind_effects,
                    yi = est,
                    V = var,
                    method = "REML",
                    test = "t",
                    random = list(~ 1|effect_size_id,
                                  ~ 1|phenotyping_center, 
                                  ~ 1|strain_fig))
  }
  
  if (output == "results") {
    df_model <- data.frame(est = model$beta[1],
                           var = model$se ^ 2,
                           lower = model$ci.lb,
                           upper = model$ci.ub,
                           phenotyping_center = "Mean",
                           strain_fig = "ES")
    
    ind_effects %>% 
      bind_rows(df_model) %>% 
      mutate(est_type = measure,
             trait_name = chosen_trait,
             centre_and_strain = factor(paste0(phenotyping_center,
                                               "\n",
                                               strain_fig),
                                        levels = rev(c("CCP-IMG\nNCrl",
                                                       "HMGU\nNCrl",
                                                       "HMGU\nNTac",
                                                       "JAX\nNJ",
                                                       "MRC H\nNTac",
                                                       "TCP\nNCrl",
                                                       "Mean\nES"))))
    
  } else if (output == "heterogeneity") {
    if (measure == "KU_delta") {
      sampling_variance <- 
        model$sigma2[2] * vtilde
      
      # from Higgins - https://doi.org/10.1002/sim.1186
      av_sv <- 
        sum(1 / sampling_variance) / 
        sum((1 / sampling_variance) ^ 2) * sum(1 / sampling_variance) ^ 2 / 
        (sum(1 / sampling_variance) * (length(sampling_variance) - 1))
      
      denominator <-
        av_sv + model$sigma2[1] + model$sigma2[3] + model$sigma2[4]
      
      tibble(trait_name_1 = chosen_trait,
             trait_name_2 = NA,
             est_type = measure) %>% 
        add_column(i2_total = (model$sigma2[1] + model$sigma2[3] + model$sigma2[4]) * 100 /
                     denominator,
                   i2_effect_size_id = model$sigma2[1] * 100 / denominator,
                   i2_phenotyping_center = model$sigma2[3] * 100 / denominator,
                   i2_strain_fig = model$sigma2[4] * 100 / denominator) %>% 
        clean_names()
    } else {
      tibble(trait_name_1 = chosen_trait,
             trait_name_2 = NA,
             est_type = measure) %>% 
        add_column(as_tibble(t(i2_ml(model)))) %>% 
        clean_names()
    }
  }
}

process.cor_effects <- 
  function(chosen_trait_1 = "fat_mass",
           chosen_trait_2 = "heart_weight",
           output = "results") {
    
    df_raw_cor <-
      df_raw %>% 
      filter(trait_name %in% c(chosen_trait_1,
                               chosen_trait_2), 
             phenotyping_center %in% c("CCP-IMG",
                                       "HMGU",
                                       "JAX",
                                       "MRC H",
                                       "TCP")) %>% 
      pivot_wider(id_cols = c(specimen_id,
                              strain_fig,
                              phenotyping_center,
                              sex),
                  names_from = trait_name) %>%
      clean_names() %>% 
      drop_na() %>% 
      group_by(strain_fig,
               phenotyping_center,
               sex) %>% 
      group_modify(~ correlate(.x)) %>% 
      drop_na(all_of(chosen_trait_2)) %>% 
      ungroup() %>%
      left_join(df_raw %>% 
                  filter(trait_name %in% c(chosen_trait_1,
                                           chosen_trait_2), 
                         phenotyping_center %in% c("CCP-IMG",
                                                   "HMGU",
                                                   "JAX",
                                                   "MRC H",
                                                   "TCP")) %>% 
                  pivot_wider(id_cols = c(specimen_id,
                                          strain_fig,
                                          phenotyping_center,
                                          sex),
                              names_from = trait_name) %>%
                  clean_names() %>% 
                  drop_na() %>% 
                  group_by(strain_fig,
                           phenotyping_center,
                           sex) %>% 
                  summarise(n = n())) %>% 
      select(- all_of(chosen_trait_1)) %>% 
      rename(r_est = chosen_trait_2) %>% 
      pivot_wider(id_cols = c(strain_fig,
                              phenotyping_center),
                  names_from = sex,
                  values_from = c(r_est,
                                  n))
    
    df_effects_cor <-
      df_raw_cor %>% 
      left_join(df_raw_cor %>% 
                  group_by(phenotyping_center,
                           strain_fig) %>% 
                  reframe(orchaRd::cor_diff(cor1 = na.omit(r_est_male),
                                            cor2 = na.omit(r_est_female),
                                            n1 = na.omit(n_male),
                                            n2 = na.omit(n_female)))) %>% 
      rename(delta_zr_est = zr_diff,
             delta_zr_var = var_zr_diff) %>% 
      mutate(delta_zr_upper = delta_zr_est + qt(0.975, 
                                                n_male + n_female - 2) * sqrt(delta_zr_var),
             delta_zr_lower = delta_zr_est - qt(0.975, 
                                                n_male + n_female - 2) * sqrt(delta_zr_var)) %>% 
      rowid_to_column("effect_size_id")
    
    model <-
      rma.mv(data = df_effects_cor,
             yi = delta_zr_est,
             V = delta_zr_var,
             test = "t",
             random = list(~ 1|effect_size_id,
                           ~ 1|phenotyping_center, 
                           ~ 1|strain_fig))
    
    if (output == "results") {
      df_model <- data.frame(delta_zr_est = model$beta[1],
                             delta_zr_lower = model$ci.lb,
                             delta_zr_upper = model$ci.ub,
                             phenotyping_center = "Mean",
                             strain_fig = "ES")
      
      df_effects_cor %>% 
        bind_rows(df_model) %>% 
        mutate(centre_and_strain = factor(paste0(phenotyping_center,
                                                 "\n",
                                                 strain_fig))) %>% 
        mutate(centre_and_strain = factor(centre_and_strain,
                                          levels = rev(c("CCP-IMG\nNCrl",
                                                         "HMGU\nNCrl",
                                                         "HMGU\nNTac",
                                                         "JAX\nNJ",
                                                         "MRC H\nNTac",
                                                         "TCP\nNCrl",
                                                         "Mean\nES"))))
    } else if (output == "heterogeneity") {
      tibble(trait_name_1 = chosen_trait_1,
             trait_name_2 = chosen_trait_2,
             est_type = "delta_zr") %>% 
        add_column(as_tibble(t(i2_ml(model)))) %>% 
        clean_names()
    }
  }

# visualisation functions ----
caterpillar.custom <- 
  function(chosen_trait = "fat_mass",
           measure = "KU_delta") {
    
    df <-
      process.ind_effects(chosen_trait = chosen_trait,
                          measure = measure)
    
    plot <-
      df %>% 
      ggplot(aes(y = centre_and_strain,
                 x = est,
                 xmax = upper,
                 xmin = lower,
                 shape = strain_fig,
                 col = phenotyping_center,
                 size = as.factor(phenotyping_center == "Mean"))) +
      geom_pointrange() +
      geom_vline(xintercept = 0,
                 linetype = "dotted") +
      annotate(geom = "label",
               # x = df$est,
               x = 0,
               y = as.numeric(df$centre_and_strain) - 0.28,
               label = paste0(round(df$est, 
                                    2),
                              " (",
                              round(df$lower, 
                                    2),
                              ", ",
                              round(df$upper, 
                                    2),
                              ")"),
               size = 2.4,
               label.size = 0,
               fill = "white",
               col = "black") +
      scale_shape_manual(values = c(18:15)) +
      scale_size_manual(values = c(0.5,
                                   0.7)) +
      theme_classic() +
      theme(legend.position = "none",
            axis.text.y = element_blank(),
            axis.title.y = element_blank(),
            plot.tag.position = c(0.15, 
                                  0.98))
    
    lab_x <- 
      case_when(measure == "ROM" ~ "lnRR",
                measure == "VR" ~ "lnVR",
                measure == "SK_delta" ~ "&Delta;*sk*",
                measure == "KU_delta" ~ "&Delta;*ku*")
    
    if (chosen_trait %in% c("fat_mass",
                            "heart_weight")) {
      lim_x <-
        case_when(measure == "ROM" ~ 0.5,
                  measure == "VR" ~ 1,
                  measure == "SK_delta" ~ 3,
                  measure == "KU_delta" ~ 45)
    } else {
      lim_x <-
        case_when(measure == "ROM" ~ 0.5,
                  measure == "VR" ~ 0.5,
                  measure == "SK_delta" ~ 1,
                  measure == "KU_delta" ~ 6)
    }
    
    plot +
      labs(x = lab_x) +
      scale_x_continuous(limits = c(- lim_x * 1.02, 
                                    lim_x * 1.02),
                         breaks = c(- lim_x, 
                                    0, 
                                    lim_x)) +
      theme(axis.title.x = ggtext::element_markdown(face = "italic"))
  }

ridgeline.custom <- function(chosen_trait = "fat_mass") {
  
  processed_data <-
    df_raw %>% 
    filter(trait_name == chosen_trait,
           phenotyping_center %in% c("CCP-IMG",
                                     "HMGU",
                                     "JAX",
                                     "MRC H",
                                     "TCP")) %>% 
    add_row(phenotyping_center = "Mean",
            strain_fig = "ES") %>% 
    mutate(centre_and_strain = factor(paste0(phenotyping_center,
                                             "\n",
                                             strain_fig))) %>% 
    mutate(centre_and_strain = factor(centre_and_strain,
                                      levels = rev(c("CCP-IMG\nNCrl",
                                                     "HMGU\nNCrl",
                                                     "HMGU\nNTac",
                                                     "JAX\nNJ",
                                                     "MRC H\nNTac",
                                                     "TCP\nNCrl",
                                                     "Mean\nES"))),
           value_s = scale(value)[, 1])
  
  sample_sizes <-
    processed_data %>% 
    count(centre_and_strain,
          sex) %>% 
    slice(-1) %>% 
    add_row(centre_and_strain = factor("Mean\nES"),
            processed_data %>% 
              filter(!is.na(sex)) %>% 
              count(sex)) %>% 
    pivot_wider(id_cols = centre_and_strain,
                names_from = sex,
                values_from = n) %>% 
    mutate(label = paste0("Nf = ",
                          female,
                          ", Nm = ",
                          male)) %>% 
    arrange(centre_and_strain)
  
  processed_data %>% 
    ggplot(aes(x = value_s,
               y = centre_and_strain,
               fill = sex,
               linetype = sex)) +
    stat_slab(scale = 0.7, 
              alpha = 0.4,
              linewidth = 0.6,
              col = "black") +
    scale_fill_manual(values = c("white",
                                 "black")) +
    scale_linetype_manual(values = c("solid",
                                     "dashed")) +
    labs(x = paste0(str_to_sentence(str_replace_all(chosen_trait,
                                                    "_",
                                                    " ")),
                    "\n(scaled)"),
         y = "Phenotyping centre and mice strain") +
    annotate(geom = "text",
             x = mean(range(processed_data$value_s,
                            na.rm = T)),
             y = as.numeric(sample_sizes$centre_and_strain) - 0.15,
             label = sample_sizes$label,
             size = 2.3) +
    theme_classic() +
    theme(legend.position = "none",
          axis.title.x = element_text(size = 12, 
                                      margin = margin(t = 0.2,
                                                      unit = "cm")),
          axis.title.y = element_text(size = 12, 
                                      margin = margin(r = 0.2,
                                                      unit = "cm")),
          axis.text.x = element_text(size = 10),
          axis.text.y = element_text(size = 10),
          plot.tag.position = c(0.53, 
                                0.98))
}

cor.caterpillar.custom <- 
  function(chosen_trait_1 = "fat_mass",
           chosen_trait_2 = "heart_weight") {
    
    df <-
      process.cor_effects(chosen_trait_1 = chosen_trait_1,
                          chosen_trait_2 = chosen_trait_2)
    
    df %>% 
      ggplot(aes(y = centre_and_strain,
                 x = delta_zr_est,
                 xmax = delta_zr_upper,
                 xmin = delta_zr_lower,
                 shape = strain_fig,
                 col = phenotyping_center,
                 size = as.factor(phenotyping_center == "Mean"))) +
      geom_pointrange() +
      geom_vline(xintercept = 0,
                 linetype = "dotted") +
      annotate(geom = "label",
               # x = df$delta_zr_est,
               x = 0,
               y = as.numeric(df$centre_and_strain) - 0.15,
               label = paste0(round(df$delta_zr_est, 
                                    2),
                              " (",
                              round(df$delta_zr_lower, 
                                    2),
                              ", ",
                              round(df$delta_zr_upper, 
                                    2),
                              ")"),
               size = 2.4,
               label.size = 0,
               fill = "white",
               col = "black") +
      labs(y = "Phenotyping centre and mice strain",
           x = "&Delta;*Zr*", 
           shape = "Strain") +
      scale_x_continuous(limits = c(-1, 
                                    1),
                         breaks = c(-1, 
                                    0, 
                                    1)) +
      scale_shape_manual(values = c(18:15)) +
      scale_size_manual(values = c(0.6,
                                   0.8)) +
      theme_classic() +
      theme(legend.position = "none",
            axis.title.x = ggtext::element_markdown(size = 12, 
                                                    margin = margin(t = 0.2,
                                                                    unit = "cm")),
            axis.title.y = element_text(size = 12,
                                        margin = margin(r = - 0.1,
                                                        unit = "cm")),
            axis.text.x = element_text(size = 10),
            axis.text.y = element_text(size = 10),
            plot.tag.position = c(0.3, 
                                  0.99))
  }

cor.plot.custom <- 
  function(chosen_trait_1 = "fat_mass",
           chosen_trait_2 = "heart_weight",
           chosen_lims = c(-3, 5)) {
    df_cor <-
      df_raw %>% 
      filter(trait_name %in% c(chosen_trait_1,
                               chosen_trait_2), 
             phenotyping_center %in% c("CCP-IMG",
                                       "HMGU",
                                       "JAX",
                                       "MRC H",
                                       "TCP")) %>% 
      pivot_wider(id_cols = c(specimen_id,
                              strain_fig,
                              phenotyping_center,
                              sex),
                  names_from = trait_name) %>%
      clean_names() %>% 
      drop_na() %>% 
      mutate(centre_and_strain = factor(paste0(phenotyping_center,
                                               strain_fig))) %>% 
      mutate(trait_1_s = scale(get(chosen_trait_1))[,1],
             trait_2_s = scale(get(chosen_trait_2))[,1])
    
    sample_sizes <-
      df_cor %>% 
      count(centre_and_strain,
            sex) %>% 
      pivot_wider(id_cols = centre_and_strain,
                  names_from = sex,
                  values_from = n) %>% 
      mutate(label = paste0("Nf = ",
                            female,
                            ", Nm = ",
                            male)) %>% 
      arrange(desc(centre_and_strain))
    
    plot_list <- list()
    
    for (i in 1:length(levels(df_cor$centre_and_strain))) {
      level_i <- sort(levels(df_cor$centre_and_strain))[i]
      
      plot <-
        df_cor %>% 
        filter(centre_and_strain == level_i) %>% 
        ggplot(aes(x = trait_1_s,
                   y = trait_2_s,
                   shape = sex,
                   linetype = sex)) +
        geom_point(alpha = 0.008) +
        geom_abline(intercept = 0,
                    slope = 1,
                    linewidth = 0.5,
                    linetype = "dotted") +
        geom_smooth(method = "lm",
                    se = F,
                    col = "black") +
        scale_shape_manual(values = c(3, 
                                      4)) +
        scale_linetype_manual(values = c("solid",
                                         "dashed")) +
        scale_x_continuous(limits = chosen_lims) +
        scale_y_continuous(limits = chosen_lims) +
        labs(x = paste0(str_to_sentence(str_replace_all(chosen_trait_1, 
                                                        "_", 
                                                        " ")),
                        "\n(scaled)"),
             y = paste0(str_to_sentence(str_replace_all(chosen_trait_2, 
                                                        "_", 
                                                        " ")),
                        " (scaled)")) +
        annotate(geom = "label",
                 x = mean(chosen_lims),
                 y = 4.5,
                 label = sample_sizes$label[i],
                 size = 2.4,
                 label.size = 0) +
        theme_classic() +
        theme(legend.position = "none",
              plot.tag.position = c(0.05, 
                                    0.91),
              axis.title.x = element_text(size = 12, 
                                          margin = margin(t = 0.2,
                                                          unit = "cm")),
              axis.title.y = element_text(size = 12, 
                                          margin = margin(r = 0.2,
                                                          unit = "cm")),
              axis.text.x = element_text(size = 10),
              axis.text.y = element_text(size = 10))
      
      
      if (i != 6) {
        plot <-
          plot +
          theme(axis.title.x = element_blank(),
                axis.text.x = element_blank(),
                axis.line.x = element_blank(),
                axis.ticks.x = element_blank())
      }
      
      plot_list[[i]] <- plot
    }
    
    return(plot_list)
  }
```


# Simulations
## Extended simulation details
We conducted Monte-Carlo simulations to evaluate bias and variance estimation for our new effect sizes $\Delta sk$, $\Delta ku$, and $\Delta Zr$. For $\Delta sk$ and $\Delta ku$ we simulated independent samples for two groups from Pearson distributions with known moments using the *rpearson* function from the PearsonDS R package (vers. 1.3.2 [1]). We conducted two simulations: 1) first by changing skewness between groups that involved moderate departures from normality (group-specific skewness, $sk \in \{-1, -0.5, 0, 0.5, 1\}$ with kurtosis fixed at 3) and 2) by holding skewness constant ($sk$ = 0) while manipulating kurtosis, $ku \in \{2.5, 3, 4, 5, 6\}$. In all cases, we simulated scenarios where: (i) the variance between each group was the same ($\sigma^2_{2}$ = $\sigma^2_{1}$ = 1) or different ($2\sigma^2_{2}$ versus $\sigma^2_{1}$); (ii) the mean between the two groups was the same ($\mu_{2}$ = $\mu_{1}$ = 0) or different ($\mu_{2}$ = 5, $\mu_{1}$ = 0). For simplicity we assumed equal sample sizes between groups with sample size varying from $n \in \{10, 20, \dots, 100, 150, 500\}$. We created all unique combinations of the above scenarios resulting in 1200 independent scenarios (when considering each of the 100 scenarios at each sample size, see examples in @sec-skewness and @sec-kurtosis). We estimated $\Delta sk$ and $\Delta ku$ for each scenario using formulas for within-group sample skewness with small-sample correction (Eq. 1 in main manuscript) and excess kurtosis with small-sample correction (Eq. 3 in main manuscript) to estimate point estimates. To estimate associated sampling variance for $\Delta sk$ and $\Delta ku$ we used the analytical variance estimators derived here and an associated re-sampling (jackknife) approach to compute group sampling variances separately followed by pooling.  Importantly, our simulations assume no correlation between groups. 

For $\Delta Zr$ simulations, we simulated two groups each containing two variables with known correlations within each group.  For $\Delta Zr$ we drew bivariate normal data with target within-group correlations $r \in \{-0.8, -0.4, -0.2, 0, 0.2, 0.4, 0.6, 0.8\}$ using the *mvnorm* function in the MASS package of R (vers. 7.3.61, [2]). Marginals were standard normal and group sizes varied from $n \in \{10, 20, \dots, 100, 150, 500\}$. Again, we created all unique combinations of scenarios resulting in 768 unique scenarios. We estimated $\Delta Zr$ using Fisher’s Z transformation $Zr$ and calculating $\Delta Zr$ as the difference of $Zr$ across groups (Eqs. 9–11 in main manuscript). Sampling variance for $\Delta Zr$ was approximated used the standard analytic variance $\frac{1}{n-3}$ per group (summed; Eq. 10 in main manuscript) and a jackknife approach. Again, we assumed no correlation between our groups. 

Across all simulations we conducted 2,500 replicates of each scenario. Performance metrics were (a) bias of the point estimator (mean estimate minus truth) (@eq-bias), (b) relative bias of the sampling-variance estimator (@eq-relbias), (c) coverage (95%) and (d) Monte-Carlo standard errors (MCSEs) (@eq-mcse). 

$$
\text{Bias}(\hat{\theta}_{mu}) = \frac{\sum_{i=1}^{n_{\text{sim}}} \hat{\theta}_i}{n_{\text{sim}}} - \theta
$$ {#eq-bias}


where $\theta_{mu}$ is the true overall mean effect (i.e., the true $\Delta sk$, $\Delta ku$, and $\Delta Zr$ between groups), and $\hat{\theta}_i$ is the effect estimate from simulation $i$. 

We calcuated coverage as the proportion of 95% confidence intervals (CIs) that contained the true effect size across all simulations for each scenario. 

To understand bias in the sampling variance, we computed the relative bias for the effect measure as:

$$
\text{Relative Bias}(\hat{\theta}_{SV}) = \left( \frac{\mathbb{E}[\hat{SV}] - \hat{\theta}^2}{\hat{\theta}^2}\right) \cdot 100\% 
$$ {#eq-relbias}

where $\hat{\theta}^2$ is the estimated sampling variance for the effect measure and $\mathbb{E}[\hat{SV}]$ is the expected value of the sampling variance. Relative bias can be calculated using different combinations of estimates. For example, we can use $\hat{\theta}^2$ as the demoninator from a point estimate taken from the small sample correction for $\Delta sk$, $\Delta ku$, or $\Delta Zr$ or from the jackknife estimate. Alternatively, we can use the jackknife estimate for $\mathbb{E}[\hat{SV}]$ across each simulation. We created each of the four combinations for our performance measures. For each of our performance measures we also computed the Monte Carlo Standard Errors (MCSE) of the estimated bias with

$$
\text{MCSE} = \sqrt{\frac{S^2_{\hat{\theta}}}{n_{\text{sim}}}}
$$ {#eq-mcse}

where $S^2_{\hat{\theta}}$ is the sample variance of the estimated effects across simulations and $n_{\text{sim}}$ is the number of simulations.

## Extended simulation results

```{r}
#| label: sim_results
#| echo: false
#| message: false

# Load in the simulation results
sk <- readRDS("./output/result_skewness.rds")
ku <- readRDS("./output/result_kurt.rds")
cor <- readRDS("./output/result_cor.rds")

# Summarise the means for all mcse columns
sk_mcse <- 
  sk %>% 
  summarise(across(starts_with("mcse"),
                   function(x) mean(x, 
                                    na.rm = TRUE)))
ku_mcse <- 
  ku %>% 
  summarise(across(starts_with("mcse"),
                   function(x) mean(x,
                                    na.rm = TRUE)))
cor_mcse <- 
  cor %>% 
  summarise(across(starts_with("mcse"),
                   function(x) mean(x, 
                                    na.rm = TRUE)))  

# Lets find out what is the best estimator. Mean Squared Bias (MSB)
result_kurt_least_bias <-
  ku %>%
  summarise(across(starts_with("bias_"),
                   ~ sum(.x^2,
                         na.rm = TRUE),
                   .names = "ms_bias_{col}")) %>%
  t() %>%
  data.frame() %>%
  arrange() 

colnames(result_kurt_least_bias) <- "MSBE"

result_skew_least_bias <-
  sk %>%
  summarise(across(starts_with("bias_"),
                   ~ sum(.x^2,
                         na.rm = TRUE),
                   .names = "ms_bias_{col}")) %>%
  t() %>%
  data.frame() %>%
  arrange() 

colnames(result_skew_least_bias) <- "MSBE"

result_cor_least_bias <-
  cor %>%
  summarise(across(starts_with("bias_"),
                   ~ sum(.x^2,
                         na.rm = TRUE),
                   .names = "ms_bias_{col}")) %>%
  t() %>%
  data.frame() %>%
  arrange() 

colnames(result_cor_least_bias) <- "MSBE"

# What is the bias for skewness where it's 0, or normally distributed
sk_normal <- 
  sk %>% 
  filter(skewness_g1 == 0 & skewness_g2 == 0) %>% 
  summarise(mean_bias_sk = mean(bias_sk),
            sd_bias_sk = sd(bias_sk),
            mean_bias_jack = mean(bias_sk_jack_bc),
            sd_bias_jack = sd(bias_sk_jack_bc),
            n = n())

# What is the bias for kurtosis where it's 3, or normally distributed
ku_normal <- 
  ku %>% 
  filter(kurtosis_g1 == 3 & kurtosis_g2 == 3) %>% 
  summarise(mean_bias_ku = mean(bias_ku),
            sd_bias_ku = sd(bias_ku),
            mean_bias_jack = mean(bias_ku_jack_bc),
            sd_bias_jack = sd(bias_ku_jack_bc),
            n = n())

# Lets create a new columns describing the average deviation of both distributions from nomrality. 
sk <- 
  sk %>%
  mutate(dev_skew = abs((skewness_g1 - 0) + (skewness_g2 - 0)))

ku <- 
  ku %>%
  mutate(dev_kurt = abs((kurtosis_g1 - 3) + (kurtosis_g2 - 3)),
         var_diff = as.factor(abs((variance_g1 - variance_g2))),
         mean_diff = as.factor(abs((mean_g1 - mean_g2))))

# Plots
sk_why <-
  ggplot(sk,
         aes(y = bias_sk,
             x = dev_skew,
             fill = n,
             colour = n)) + 
  geom_point(size = 2) + 
  geom_hline(yintercept = 0, 
             linetype = "dashed",
             color = "black") +
  labs(y = TeX("$Bias_{\\Delta \\textit{sk}}$ (small-sample estimator)"), 
       x = TeX("Absolute deviation from normality $ \\left( \\left| ( \\textit{sk}_{g1} - 0 ) + (\\textit{sk}_{g2} - 0 ) \\right| \\right)$"), 
       fill = "Sample size",
       colour = "Sample size") +
  scale_fill_viridis_c() +
  scale_colour_viridis_c() +
  theme_classic()

ku_why <- 
  ggplot(ku,
         aes(y = bias_ku,
             x = dev_kurt,
             fill = n,
             colour = n)) +
  geom_point(size = 2) +
  geom_hline(yintercept = 0,
             linetype = "dashed",
             color = "black") +
  labs(y = TeX("$Bias_{\\Delta \\textit{ku}}$ (small-sample estimator)"), 
       x = TeX("Absolute deviation from normality $ \\left( \\left| (\\textit{ku}_{g1} - 3 ) + (\\textit{ku}_{g2} - 3 ) \\right| \\right)$"), 
       fill = "Sample Size",
       colour = "Sample Size") +
  theme_classic() +
  scale_fill_viridis_c() +
  scale_colour_viridis_c()

plot_why <-
  sk_why +
  ku_why + 
  plot_layout(nrow = 2) +
  plot_annotation(tag_levels = "A")

# ggsave("./output/figs/plot_why.png",
#        plot = plot_why,
#        bg = "white",
#        dpi = 600,
#        width = 7,
#        height = 7,
#        units = "in")
```

In all cases, we found the Monte Carlo Sampling Error (MCSEs) to be low for all our performance metrics ($\Delta sk$: range of MCSEs, `r round(min(sk_mcse), digits = 3)` to `r round(max(sk_mcse), digits = 3)`; $\Delta ku$: range of MCSEs, `r round(min(ku_mcse), digits = 3)` to `r round(max(ku_mcse), digits = 3)`; $\Delta Zr$: range of MCSEs, `r round(min(cor_mcse), digits = 3)` to `r round(max(cor_mcse), digits = 3)`). 

#### Evaluating $\Delta sk$, $\Delta ku$, and $\Delta Zr$ point estimators

Across all simulations, $\Delta sk$, $\Delta ku$, and $\Delta Zr$ point estimators exhibited small sample bias with less than 20-30 samples, except for $\Delta ku$, which continued to show this bias through to n ~ 50-60, indicating effect sizes involving kurtosis are more challenging to estimate (see Fig 2). Regardless, small sample biases were moderate, and there was rarely a consistent over or under-estimation in point estimates across the scenarios evaluated (see Fig 2). 

As expected, both the analytical and jackknife estimates for $\Delta sk$ and $\Delta ku$ had minimal bias when distributions were normally distributed (Skewness of both groups = 0; Analytical bias= `r round(sk_normal$mean_bias_sk, digits = 3)` with SD = `r round(sk_normal$sd_bias_sk, digits = 3)` & Jackknife = `r round(sk_normal$mean_bias_jack, digits = 3)` with SD = `r round(sk_normal$sd_bias_jack, digits = 3)`; Kurtosis of both groups = 3; Analytical bias=`r round(ku_normal$mean_bias_ku, digits = 3)` with SD = `r round(ku_normal$sd_bias_ku, digits = 3)` & Jackknife = `r round(ku_normal$mean_bias_jack, digits = 3)` with SD = `r round(ku_normal$sd_bias_jack, digits = 3)`), but bias in both seemed to change in complex ways that suggest challenges in estimating moments (see S2 Fig). This did not appear to be related to differences in group means or variances as bias patterns were similar across these conditions (see S3 Fig), but rather the sampling distributions being skewed (see "Coverage" below).

Importantly, bias-corrected jackknife estimates reduced the small-sample bias relative to analytical bias corrected-moment estimators (see S1 Fig; $\Delta sk$ Mean Square Bias (MSB): Jackknife: `r round(result_skew_least_bias$MSBE[rownames(result_skew_least_bias)=="ms_bias_bias_sk_jack_bc"], digits = 3)`, Analytical: `r round(result_skew_least_bias$MSBE[rownames(result_skew_least_bias) =="ms_bias_bias_sk"], digits = 3)`; $\Delta ku$ Mean Square Bias (MSB): Jackknife: `r round(result_kurt_least_bias$MSBE[rownames(result_kurt_least_bias) =="ms_bias_bias_ku_jack_bc"], digits = 3)`, Analytical: `r round(result_kurt_least_bias$MSBE[rownames(result_kurt_least_bias) =="ms_bias_bias_ku"], digits = 3)`; $\Delta cor$ Mean Square Bias (MSB): Jackknife: `r round(result_cor_least_bias$MSBE[rownames(result_cor_least_bias)=="ms_bias_bias_jack_d_cor"], digits = 3)`, Analytical: `r round(result_cor_least_bias$MSBE[rownames(result_cor_least_bias)=="ms_bias_bias_d_cor"], digits = 3)`). 

#### Evaluating sampling variance estimators for $\Delta sk$, $\Delta ku$, and $\Delta Zr$

In contrast to point estimators, the effectiveness of sampling variance estimators for $\Delta sk$, $\Delta ku$, and $\Delta Zr$ varied. Analytical sampling variance formulas for $\Delta sk$ and $\Delta ku$ were consistently biased (see S4 Fig). Jackknife resampling, when combined with analytical point estimates (see S4 Fig D, E), improved performance. Under these conditions, estimators performed well when n > 50. In contrast, the performance of sampling variance estimators for $\Delta Zr$ was best when using the analytical formulas for both the point estimator and it's associated sampling variance (see S4 Fig I).

#### 95% Coverage

Coverage was generally close to nominal levels (95%) for $\Delta sk$ and $\Delta Zr$, but slighly poorer when sample sizes were small (< n = 20) (see S5 Fig). For $\Delta Zr$, coverage was close to nominal levels across all sample sizes when using the analytical sampling variance estimator (see S5 Fig). However, coverage was poor for $\Delta ku$ across many scenarios no matter what estimator was used (see S5 Fig). Unintuitively, coverage for $\Delta ku$ was often worse as sample sizes were larger (see S5 Fig), driven by the fact that point estimates were more accurately estimated with large sample sizes, but the sampling distribution became highly skewed, impacting coverage (see S6 Fig). At small sample sizes $\Delta ku$ was estimated poorly when true $\Delta ku$ was high, leading to non-skewed distributions with good coverage. In contrast, large sample sizes improved point estimation of $\Delta ku$  when true differences existed, but the sampling distribution became highly skewed leading to poor coverage (see S6 Fig). As a sensitivity analysis, we also evaluated coverage when using bootstrap resampling to estimate sampling variances for all effect sizes. Results were qualitatively similar. 

## Summary
In light of these simulation results, we suggest pairing the formula-based point estimators for skewness (Eq. 1 in main manuscript) and kurtosis (Eq. 3 in main manuscript) with jackknife standard errors for $\Delta sk$ and $\Delta ku$. For $\Delta Zr$, the standard analytic variance is recommended (Eqns. 9-12 in main manuscript). This choice balances efficiency under normality with robustness to realistic deviations from it, and aligns with our broader guidance to avoid very small group sizes for these statistics. Given the challenges in estimating $\Delta ku$, and the poor properties of its sampling variance, we recommend weighted meta-analytic models using sample size instead of sampling variance. 

# Equations and custom functions to calculate effect sizes

## Skewness

Following Joanes & Gill (1998).  


$$
sk = \frac{\frac{1}{n} \sum_{i = 1}^{n}(x_{i} - \bar{x}) ^ 3}{[\frac{1}{n} \sum_{i = 1}^{n}(x - \bar{x}) ^ 2] ^ \frac{3}{2}}
\frac{\sqrt{n (n - 1)}}{n - 2}
$$
$$
s^2_{sk} = \frac{6n(n - 1)}{(n - 2)(n + 1)(n + 3)}
$$

$$
\Delta sk = sk_{1} - sk_{2}
$$

$$
s^2_{\Delta sk} = s^2_{sk_1} + s^2_{sk_2} - 2 \rho_{sk} s_{sk_1} s_{sk_2}
$$

## Kurtosis 

Following Joanes & Gill (1998).  

$$
ku = \frac{n (n + 1) (n - 1)}{(n - 2)(n - 3)} 
\frac{\sum_{i = 1}^{n}(x_{i} - \bar{x}) ^ 4}
{[\sum_{i = 1}^{n}(x_{i} - \bar{x}) ^ 2]^ 2} -
\frac{3(n - 1) ^ 2}{(n - 2)(n - 3)}
$$
$$
s^2_{ku} = \frac{24 n (n - 1) ^ 2}{(n - 3)(n - 2)(n + 3)(n + 5)} 
$$

$$
\Delta ku = ku_{1} - ku_{2}
$$

$$
s^2_{\Delta ku} = s^2_{ku_1} + s^2_{ku_2} - 2 \rho_{ku} s_{ku_1} s_{ku_2}
$$

## Zr

Following Hedges & Olkin (1985).  

$$
Zr =  \frac{ln(\frac{1 + r}{1 - r})}{2}
$$

$$
s^2_{Zr} = \frac{1}{n - 3}
$$
$$
\Delta Zr = Zr_{1} - Zr_{2}
$$

$$
s^2_{\Delta Zr} = s^2_{Zr_1} + s^2_{Zr_2} -2 \rho_{Zr} s_{Zr_1} s_{Zr_2}
$$

# Data loading and preparation

We use data from the International Mouse Phenotyping Consortium (IMPC, version 18.0; [3]; http://www.mousephenotype.org/).

```{r data}
# raw data ----
df_raw <- 
  read_csv("mice_data_sample.csv") %>% 
  # small adjustments to make plots more readable:
  mutate(phenotyping_center = 
           ifelse(phenotyping_center == "MRC Harwell",
                  "MRC H",
                  phenotyping_center),
         strain_fig = case_when(strain_accession_id == "MGI:2159965" ~ 
                                  "N",
                                strain_accession_id == "MGI:2683688" ~ 
                                  "NCrl",
                                strain_accession_id == "MGI:2164831" ~ 
                                  "NTac",
                                strain_accession_id == "MGI:3056279" ~ 
                                  "NJ",
                                strain_accession_id == "MGI:2160139" ~ 
                                  "NJcl"))

df_raw_wide <-
  df_raw %>% 
  select(- strain_accession_id) %>% 
  pivot_wider(id_cols = c(specimen_id,
                          trait_name,
                          phenotyping_center,
                          strain_fig),
              names_from = sex,
              values_from = value)

df_meta_analysed <- # takes 30s to a minute to run
  df_raw %>% 
  group_by(sex,
           trait_name,
           phenotyping_center,
           strain_fig) %>% 
  summarize(mean = mean(value,
                        na.rm = T),
            sd = sd(value,
                    na.rm = T),
            n = n()) %>% 
  pivot_wider(id_cols = c(trait_name,
                          phenotyping_center,
                          strain_fig),
              names_from = sex,
              values_from = c(mean:n)) %>% 
  bind_cols(calc.effect(., m = "ROM")) %>% # lnRR
  bind_cols(calc.effect(., m = "CVR")) %>% # lnCVR
  bind_cols(calc.effect(., m = "VR")) %>%  # lnVR
  left_join(df_raw_wide %>% 
              group_by(trait_name,
                       phenotyping_center,
                       strain_fig) %>% 
              reframe(orchaRd::moment_effects(x1 = na.omit(male),
                                              x2 = na.omit(female),
                                              type = "skew"))) %>% 
  left_join(df_raw_wide %>% 
              group_by(trait_name,
                       phenotyping_center,
                       strain_fig) %>% 
              reframe(orchaRd::moment_effects(x1 = na.omit(male),
                                              x2 = na.omit(female),
                                              type = "kurt"))) %>% 
  rename(SK_delta_est = d_skew,
         SK_delta_var = d_skew_v,
         KU_delta_est = d_kurt,
         KU_delta_var = d_kurt_v) %>% 
  mutate(n_total = n_female + n_male,
         prop_females = n_female / (n_female + n_male)) %>% 
  select(trait_name,
         phenotyping_center,
         strain_fig,
         n_total,
         n_male,
         n_female,
         prop_females,
         ROM_est,
         ROM_var,
         CVR_est,
         CVR_var,
         VR_est,
         VR_var,
         SK_delta_est,
         SK_delta_var,
         KU_delta_est,
         KU_delta_var) %>% 
  mutate(ROM_upper = ROM_est + qt(0.975, 
                                  n_total - 1) * sqrt(ROM_var),
         ROM_lower = ROM_est - qt(0.975, 
                                  n_total - 1) * sqrt(ROM_var),
         CVR_upper = CVR_est + qt(0.975, 
                                  n_total - 1) * sqrt(CVR_var),
         CVR_lower = CVR_est - qt(0.975, 
                                  n_total - 1) * sqrt(CVR_var),
         VR_upper = VR_est + qt(0.975, 
                                n_total - 1) * sqrt(VR_var),
         VR_lower = VR_est - qt(0.975, 
                                n_total - 1) * sqrt(VR_var),
         SK_delta_upper = SK_delta_est + qt(0.975, 
                                            n_total - 1) * sqrt(SK_delta_var),
         SK_delta_lower = SK_delta_est - qt(0.975, 
                                            n_total - 1) * sqrt(SK_delta_var),
         KU_delta_upper = KU_delta_est + qt(0.975, 
                                            n_total - 1) * sqrt(KU_delta_var),
         KU_delta_lower = KU_delta_est - qt(0.975, 
                                            n_total - 1) * sqrt(KU_delta_var))
```

# Meta-analytical models

We then use the data from multiple phenotyping centres and mice strains to calculate average effect sizes ($\Delta sk$, $\Delta ku$, and $\Delta Zr$).

## Table A. Single variable effect sizes

```{r}
map2_dfr(.x = rep(c("fat_mass",
                    "heart_weight",
                    "glucose",
                    "total_cholesterol"),
                  each = 4),
         .y = rep(c("ROM",
                    "VR",
                    "SK_delta",
                    "KU_delta"), 
                  4),
         .f = process.ind_effects) %>% 
  dplyr::select(- c(effect_size_id,
                    sampling_error_id,
                    n0)) %>% 
  mutate(est_type = case_when(est_type == "ROM" ~ "lnRR",
                              est_type == "VR" ~ "lnVR",
                              est_type == "SK_delta" ~ "delta_sk",
                              est_type == "KU_delta" ~ "delta_ku")) %>% 
  datatable(.,
            extensions = "Buttons",
            rownames = FALSE)
```

## Table B. Correlational effect sizes

```{r}
map2_dfr(.x = c("fat_mass",
                "glucose"),
         .y = c("heart_weight",
                "total_cholesterol"),
         .f = process.cor_effects) %>% 
  mutate(relationship = rep(c("fat mass and heart weight",
                              "glucose and total cholesterol"),
                            each = 7)) %>% 
  dplyr::select(- effect_size_id) %>% 
  relocate(relationship) %>%  
  datatable(.,
            extensions = "Buttons",
            rownames = FALSE)
```

## Table C. Heterogeneity estimates

```{r}
map2_dfr(.x = rep(c("fat_mass",
                    "heart_weight",
                    "glucose",
                    "total_cholesterol"),
                  each = 4),
         .y = rep(c("ROM",
                    "VR",
                    "SK_delta",
                    "KU_delta"), 
                  4),
         .f = ~ process.ind_effects(chosen_trait = .x,
                                    measure = .y,
                                    output = "heterogeneity")) %>% 
  mutate(est_type = case_when(est_type == "ROM" ~ "lnRR",
                              est_type == "VR" ~ "lnVR",
                              est_type == "SK_delta" ~ "delta_sk",
                              est_type == "KU_delta" ~ "delta_ku")) %>% 
  bind_rows(map2_dfr(.x = c("fat_mass",
                            "glucose"),
                     .y = c("heart_weight",
                            "total_cholesterol"),
                     .f = ~ process.cor_effects(chosen_trait_1 = .x,
                                                chosen_trait_2 = .y,
                                                output = "heterogeneity"))) %>% 
  datatable(.,
            extensions = "Buttons",
            rownames = FALSE)
```

## Meta-analysis validation

Statistical validation of meta-analytic models was performed to study statistical error and bias in meta-analytic estimates, the influence of varying levels of heterogeneity, the impact of meta-analytical sample size, and the agreement between a summary-level MA and an IPD-type meta-analysis of idividual-level data. Simulations were performed along the following lines:
1. Original IMPC data was subsampled to generate datasets of varying sizes (k = 5, 10, 20, 40, 60, 80 "studies" per meta-analysis; within-study sample size of 50 or 100 individuals).
2. For each subsampled dataset, known effect size magnitude was introduced by Box-Cox transforming the data within sexes to generate known differences in skewness, or by introducing outliers to generate known differences in kurtosis. We generated three scenarions of effect-size magnitudes: small (0.3), medium (0.5), and large (1.0) for both skewness and kurtosis.
3. Meta-analytical models were fitted to each simulated dataset to estimate effect sizes, using the estimators and sampling variances recommended from the simulation study (see package `orchaRd` for details). Each simulation scenario was repeated 200 times.

See summary plots of the validations below (Figs U, V, W, X).

# Visualisations

## Scenarios explored for differences in skewness {#sec-skewness}

::: {.panel-tabset .tabset-pills .tabset-fade}

### Fig A
 
```{r}
#| label: fig-skewness-1
#| echo: false
#| 
# Plot 1 code goes here
files <- list.files("./output/figs/skewness/")
images  <- lapply(files, 
                  function(x) {
                    magick::image_read(paste0("./output/figs/skewness/", 
                                              x))
                  })

images[[1]]
```

### Fig B
```{r}
#| label: fig-skewness-2
#| echo: false
# Plot 2 code goes here
images[[2]]

```

### Fig C
```{r}
#| label: fig-skewness-3
#| echo: false
# Plot 3 code goes here
images[[3]]
```

### Fig D
```{r}
#| label: fig-skewness-4
#| echo: false
# Plot 4 code goes here
images[[4]]
```

### Fig E
```{r}
#| label: fig-skewness-5
#| echo: false
# Plot 5 code goes here
images[[5]]
```


### Fig F
```{r}
#| label: fig-skewness-6
#| echo: false
# Plot 6 code goes here
images[[6]]
```

### Fig G
```{r}
#| label: fig-skewness-7
#| echo: false
# Plot 7 code goes here
images[[7]]
```

### Fig H
```{r}
#| label: fig-skewness-8
#| echo: false
# Plot 8 code goes here
images[[8]]
``` 

### Fig I
```{r}
#| label: fig-skewness-9
#| echo: false
# Plot 9 code goes here
images[[9]]
```

### Fig J
```{r}
#| label: fig-skewness-10
#| echo: false
# Plot 10 code goes here
images[[10]]
```
:::


## Scenarios explored for differences in kurtosis {#sec-kurtosis}


::: {.panel-tabset .tabset-pills .tabset-fade}

### Fig K

```{r}
#| label: fig-kurtosis-1
#| echo: false
#| 
# Plot 1 code goes here
files <- list.files("./output/figs/kurtosis/")
images  <- lapply(files,
                  function(x) {
                    magick::image_read(paste0("./output/figs/kurtosis/",
                                              x))
                  })

images[[1]]
```

### Fig L
```{r}
#| label: fig-kurtosis-2
#| echo: false
# Plot 2 code goes here
images[[2]]

```

### Fig M
```{r}
#| label: fig-kurtosis-3
#| echo: false
# Plot 3 code goes here
images[[3]]
```

### Fig N
```{r}
#| label: fig-kurtosis-4
#| echo: false
# Plot 4 code goes here
images[[4]]
```

### Fig O
```{r}
#| label: fig-kurtosis-5
#| echo: false
# Plot 5 code goes here
images[[5]]
```

### Fig P
```{r}
#| label: fig-kurtosis-6
#| echo: false
# Plot 6 code goes here
images[[6]]
```

### Fig Q
```{r}
#| label: fig-kurtosis-7
#| echo: false
# Plot 7 code goes here
images[[7]]
```

### Fig R
```{r}
#| label: fig-kurtosis-8
#| echo: false
# Plot 8 code goes here
images[[8]]
``` 

### Fig S
```{r}
#| label: fig-kurtosis-9
#| echo: false
# Plot 9 code goes here
images[[9]]
```

### Fig T
```{r}
#| label: fig-kurtosis-10
#| echo: false
# Plot 10 code goes here
images[[10]]
```
:::

## MA validation - visualisations

### Fig U

```{r}
#| label: Fig_U
#| fig-cap: Fig U. MA validation results for skewness and kurtosis differences for MA of varying size (horizontal axis) and within-study sample size of 50 or 100 (colour). Vertical axis shows the power to detect small (0.3), medium (0.5), and large (1.0) effect sizes for kurtosis (top panel) and skewness (bottom panel).
#| echo: false
#|  
files <- list.files("./output/ma_validation/plots")
images  <- lapply(files, 
                  function(x) {
                    magick::image_read(paste0("./output/ma_validation/plots/", 
                                              x))
                  })

images[[4]]
```

### Fig V


```{r}
#| label: Fig_V
#| fig-cap: Fig V. MA validation results for skewness and kurtosis differences for MA of varying size (horizontal axis) and within-study sample size of 50 or 100 (colour). Vertical axis shows the estimation bias for small (0.3), medium (0.5), and large (1.0) effect sizes for kurtosis (top panel) and skewness (bottom panel).
#| echo: false
#|  
images[[1]]
```

### Fig W

```{r}
#| label: Fig_W
#| fig-cap: Fig W. MA validation results for skewness and kurtosis (colour-coded) differences for MA of varying heterogeneity (horizontal axis). Top panel - estimation bias of main effect size; bottom panel - mean estimated between-study variance (on log scale). Within-study sample size was set to 100 and effect size magnitude to medium (0.5). Plots show one selected trait (glucose levels).
#| echo: false
#| 
images[[2]]
```

### Fig X
```{r}
#| label: Fig_x
#| fig-cap: Fig X. Comparison of summary-level meta-analysis and individual participant data (IPD)-type meta-analysis for skewness and kurtosis differences. Top panel - main effect size estimates from both types of MA for a number of traits (shape-coded), and both effect size metrics (skeweness/kurtosis differences, colour-coded); dashed line indicates the `y~x` line. Bottom-panel - Bland-Altman plot comparing both approches. Within-study sample size was set to 100 and effect size magnitude to medium (0.5).
#| echo: false
#| 
images[[3]]
```

# Software and package versions  

```{r versions}
sessionInfo() %>% 
  pander()
```

# Reference  
1. Becker M, Klößner S. PearsonDS: Pearson distribution system. 2025. Available: https://cran.r-project.org/package=PearsonDS  
2. Venables WN, Ripley BD. Modern applied statistics with S. New York, NY: Springer New York; 2002. doi:10.1007/978-0-387-21706-2
3. Dickinson ME, Flenniken AM, Ji X, Teboul L, Wong MD, White JK, et al. High-throughput discovery of novel developmental phenotypes. Nature. 2016;537: 508–514. doi:10.1038/nature19356
```
