# Supplementary material for: New approaches to meta-analyze differences in skewness, kurtosis, and correlation
Source: PLoS Biol. 2026 Feb 13;24(2):e3003653. doi: 10.1371/journal.pbio.3003653 (PMC12928594; doi:10.1371/journal.pbio.3003653)
Supplement: Attachment — Submitted filename: response_letter_to_reviewers.docx [file pbio.3003653.s009.docx]

Sydney, 1 September 2025

Dear Dr. Roberts,

Thank you for the opportunity to submit a revised version of our manuscript ‘*Beyond sex differences in the mean: new approaches to meta-analyse differences in skewness, kurtosis, and correlation*’. We thank you and the reviewers for their helpful and insightful suggestions, which have made the manuscript stronger. Please find our responses (in black) to all comments (in grey) below, with excerpts from the manuscript in blue.

Dear Dr Pollo,

Thank you for your patience while your manuscript "Beyond sex differences in mean: meta-analysis of differences in skewness, kurtosis, and correlation" was peer-reviewed at PLOS Biology. Your manuscript has been evaluated by the PLOS Biology editors, an Academic Editor with relevant expertise, and by three independent reviewers.

You'll see that reviewer #1 is broadly positive, but focuses on the larger samples sizes needed to reliably calculate these higher moments, and feels that your statement on this in the limitations is insufficient; instead, s/he wants a much more substantial discussion of this issue, potentially with further analysis, and a justification of your n=50 threshold. Reviewer #2 is significantly more sceptical, noting that you seem to use “off the shelf” calculations in a rather simplistic way (my interpretation) that undermines his confidence in their use for meta-analysis. He suggests that some significant validation is needed before this will be acceptable. Reviewer #3 (in an attachment) is quite positive, but wonders if the need for n=50 will limit its utility for preclinical studies, and (like rev #2) asks about the provenance of the formulae used in the code.

As you will see in the reviewer reports, which can be found at the end of this email, although the reviewers find the work potentially interesting, they have also raised a substantial number of important concerns. Based on their specific comments and following discussion with the Academic Editor, it is clear that a substantial amount of work would be required to meet the criteria for publication in PLOS Biology. However, given our and the reviewers’ interest in your study, we would be open to inviting a comprehensive revision of the study that thoroughly addresses all the reviewers' comments. Given the extent of revision that would be needed, we cannot make a decision about publication until we have seen the revised manuscript and your response to the reviewers' comments. Your revised manuscript would need to be seen by the reviewers again, but please note that we would not engage them unless their main concerns have been addressed.

Having discussed the reviews with the Academic Editor, we think you should address all of the reviewers' comments, but in particular the requests from reviewer #2 for a more thorough characterisation and validation of your approach.

In addition to these revisions, you will need to complete some formatting changes, which you will receive in a follow up email. A member of our team will be in touch with a set of requests shortly.

We appreciate that these requests represent a great deal of extra work, and we are willing to relax our standard revision time to allow you 6 months to revise your study. Please email us (plosbiology@plos.org) if you have any questions or concerns, or envision needing a (short) extension.

At this stage, your manuscript remains formally under active consideration at our journal; please notify us by email if you do not intend to submit a revision so that we may withdraw it.

**IMPORTANT - SUBMITTING YOUR REVISION**

Your revisions should address the specific points made by each reviewer. Please submit the following files along with your revised manuscript:

1. A 'Response to Reviewers' file - this should detail your responses to the editorial requests, present a point-by-point response to all of the reviewers' comments, and indicate the changes made to the manuscript.

*NOTE: In your point by point response to the reviewers, please provide the full context of each review. Do not selectively quote paragraphs or sentences to reply to. The entire set of reviewer comments should be present in full and each specific point should be responded to individually, point by point.

You should also cite any additional relevant literature that has been published since the original submission and mention any additional citations in your response.

2. In addition to a clean copy of the manuscript, please also upload a 'track-changes' version of your manuscript that specifies the edits made. This should be uploaded as a "Revised Article with Changes Highlighted " file type.

*Resubmission Checklist*

When you are ready to resubmit your revised manuscript, please refer to this resubmission checklist: <https://plos.io/Biology_Checklist>

To submit a revised version of your manuscript, please go to <https://www.editorialmanager.com/pbiology/> and log in as an Author. Click the link labelled 'Submissions Needing Revision' where you will find your submission record.

Please make sure to read the following important policies and guidelines while preparing your revision:

*Published Peer Review*

Please note while forming your response, if your article is accepted, you may have the opportunity to make the peer review history publicly available. The record will include editor decision letters (with reviews) and your responses to reviewer comments. If eligible, we will contact you to opt in or out. Please see here for more details:

<https://blogs.plos.org/plos/2019/05/plos-journals-now-open-for-published-peer-review/>

*PLOS Data Policy*

Please note that as a condition of publication PLOS' data policy (<http://journals.plos.org/plosbiology/s/data-availability>) requires that you make available all data used to draw the conclusions arrived at in your manuscript. If you have not already done so, you must include any data used in your manuscript either in appropriate repositories, within the body of the manuscript, or as supporting information (N.B. this includes any numerical values that were used to generate graphs, histograms etc.). For an example see here: <http://www.plosbiology.org/article/info%3Adoi%2F10.1371%2Fjournal.pbio.1001908#s5>

*Blot and Gel Data Policy*

We require the original, uncropped and minimally adjusted images supporting all blot and gel results reported in an article's figures or Supporting Information files. We will require these files before a manuscript can be accepted so please prepare them now, if you have not already uploaded them. Please carefully read our guidelines for how to prepare and upload this data: <https://journals.plos.org/plosbiology/s/figures#loc-blot-and-gel-reporting-requirements>

*Protocols deposition*

To enhance the reproducibility of your results, we recommend that if applicable you deposit your laboratory protocols in protocols.io, where a protocol can be assigned its own identifier (DOI) such that it can be cited independently in the future. Additionally, PLOS ONE offers an option for publishing peer-reviewed Lab Protocol articles, which describe protocols hosted on protocols.io. Read more information on sharing protocols at <https://plos.org/protocols?utm_medium=editorial-email&utm_source=authorletters&utm_campaign=protocols>

Thank you again for your submission to our journal. We hope that our editorial process has been constructive thus far, and we welcome your feedback at any time. Please don't hesitate to contact us if you have any questions or comments.

Sincerely,

Roli Roberts

Roland Roberts, PhD

Senior Editor

PLOS Biology

rroberts@plos.org

-----------------------------------------

REVIEWERS' COMMENTS:

# Reviewer #1:

This is an interesting manuscript proposing to extend meta-analyses by including measures of skewness and kurtosis and correlations of variables. The focus, here, is on a population contrast based on the binary variable sex, though as the authors argue, the same approach might be applicable to other scenarios.

I have only one major and rather general issue and I will phrase my question in a rather non-technical way. Estimating and comparing variances is more difficult than estimating means. (You need larger samples for getting reliable estimates for variances than you need for means.) The explanation that I recall from my stats teacher was: as you have the term (xi-x-bar)^2 in your equation for the variance, the squaring has the effect that it 'magnifies' both sampling variation and measurement error. Hence, the increased variability and the need for larger samples. Of course, the stats teacher also briefly mentioned skew and kurtosis, but with the caveat that with higher moments the uncertainty in your estimates increase (having ^3 and ^4 in the equation). That was the explanation I got, why -even though informative in theory-- people rarely look closely at those higher moments.

Here, the authors do two things: first, they suggest adding a sampling variance for the skewness and kurtosis. Second, in the discussion, they have a section "Limitations", where they recommend a minimum sample size of 50, as skewness and kurtosis estimates can become rather unreliable for smaller samples (tending to produce extreme values).

I think that this sentence is not sufficient. What I would like to see would be a substantially more thorough discussion of how the higher moments are affected by sampling variation and also by measurement error or bias. This might require maybe more that the analysis of one example data set (maybe some simulations or analytic treatment) and also maybe more than a rule-of-thumb threshold number of N=50, which is coming a bit out of the blue. (Not that 50 might not be a sensible number, but I would love to see a bit more, how the authors arrived at this number. Also, I wonder whether the recommendation is actually the same for skewness and kurtosis? If my rather naïve intuition regarding the problem with the higher moments is right, one might need larger samples for kurtosis than for skewness.)

R1.1: We thank the reviewer for this comment and we agree with them. We have now conducted a simulation study varying sample sizes across 100 different scenarios to evaluate the effectiveness of our estimators and associated sampling variances for differences in skewness, kurtosis and correlations between groups. We have added the results of these simulations to the revised manuscript (lines 171-222) with more details laid out in our supplementary online material on how we set these up. The reviewer makes a valid point about how we arrived at our suggested sample size of n = 50. Based on our simulations, for comparing differences in skewness and correlations between groups, n = 20-30 will be sufficient under a diversity of different situations. In contrast, differences in kurtosis, as expected, require larger sample sizes (n ~50-60) to stabilize small sample biases and estimate the sampling variance. Importantly, our simulations highlighted the need to develop a more robust method for estimating sampling variance for differences in skewness and kurtosis. As such, we have shown a Jackknife based re-sampling approach will be able to estimate the sampling variance when sample sizes are above n = 40-50 in each group.

# Reviewer #2:

[identifies himself as Andrew W Brown]

The manuscript, "Beyond sex differences in mean: meta-analysis of differences in skewness, kurtosis, and correlation," focuses on estimating and synthesizing comparisons of skewness and kurtosis between sexes in a set of mouse data, as well as comparing correlations. Comparing other aspects of distributions beyond means is important, and there is some work already on handling variance. Skew and kurtosis represent other important aspects of continuous data that are often transformably normal, which may have important considerations in biology, such when traits are under selective pressure in which data in the tails may be more likely to be affected or selected. In this way, the authors bring up an important point. However, the authors discuss these 'effect sizes' without citing or deriving the formulae. As far as I can tell, the approach was to use off-the-shelf calculations of skewness, kurtosis, and correlation and their variances, take a simple difference of the two variables, and assume that the variance of the difference is simply a function of the typical pooled variance. Whereas the central limit theorem works well for means and standard errors, other parameters may not be estimated as well. For example, consider pooling something like maximum lifespan in this way: take the maximum of males and maximum of females and substract. Yet, we know that there are unfavorable statistical properties of sample maxima. It is not obvious to me that shape parameters defining distributions converge like means, nor am I convinced that they are problematic like extrema, but that is for the authors to prove. There are also potential concerns with interpretation in the face of differences in other parameters. Consider interpreting differences in kurtosis or skew in the face of substantial heterogeneity of variance.

Together, these concerns undermine confidence in the meta-analysis of these estimates. The authors meta-analyze these estimated differences by taking the means across sites and strains. We know that means of other distributional parameters (or their differences) do not necessarily behave like means of means (the typical question in meta-analyses). Even means needed bias corrections, with Hedge's g resulting from Cohen's d. There also does not seem to be any estimates of heterogeneity that would be expected from a random effects meta-analysis.

It is also not clear that using a random effects meta-analysis is appropriate for the question, given the authors are comparing different strains and sites. Although the design heterogeneity would necessitate a random effects meta-analysis in other circumstances, it is not clear that there is a coherent distribution of effect sizes that would be appropriately represented by a random effects meta-analysis in this case. For example, the authors have not justified that pooling differences in kurtosis across strains makes scientific sense, when the kurtosis could be different across strains.

Given the authors are interested in these effect sizes in the case of meta-analysis, the authors need to characterize and validate the approach. For instance, the authors could pool all of the data into one 'true' population and empirically set the 'true' parameter values. After establishing the parent distribution, draw multiple samples from the 'true' population, estimate the parameter of interest and its variance, compare to the 'true' parameter, and repeat. Then, get some estimate of performance, like confidence interval coverage. It could be something like this (<https://doi.org/10.1038/s41366-020-0554-2>), but would need to include some aspect of meta-analysis. If using random effects meta-analysis, the data generating process that gives rise to differences across strains and sites would need to be defined. The authors have the individual-level data, and show site/strain specific distributions, but never define the overall distribution that is being compared; rather, they present only the mean ES.

A couple of other thoughts:

The results discuss individual samples and whether certain estimates cover the null, but the purpose of a meta-analysis is to pool data to answer a coherent question, so emphasizing single studies runs counter to the purpose (e.g., line 187).

R2.1: We are unsure what reviewer 2 means by "...individual samples and whether certain estimates cover the null” or why analysing individual samples to derive effect statistics and meta-analysing these “runs counter to the purpose [of meta-analysis]”. We believe that summarising individual data into effect statistics and meta-analysing these statistics is in fact consistent with meta-analysis in general, and allows for effect variability and heterogeneity to be explored using standard meta-analytic models (as an example, see Zajitschek et al. 2020). If Reviewer 2 can clarify these points we are happy to make changes that reflect their perspective.

S. R. Zajitschek, F. Zajitschek, R. Bonduriansky, R. C. Brooks, W. Cornwell, D. S. Falster, M. Lagisz, J. Mason, A. M. Senior, D. W. Noble, S. Nakagawa, Sexual dimorphism in trait variability and its eco-evolutionary and statistical implications. *eLife* **9** (2020).

The recommendation at 252 to use 50 animals for each group seems completely arbitrary. This needs to be empirically justified (e.g., as the CLT is for sample size of 30).

R2.2: We agree. We have now conducted simulations to show that, across a diversity of scenarios, using existing small-sample corrected point estimates along with a re-sampling based jackknife approach, n ~ 50 is sufficient. We have now added these simulation results to the main manuscript with extended details and results in the supplement (see R1.1).

Figures indicate data are scaled, but no information is given about the scaling.

R2.3: We have now added details on how scaling was conducted to the legend of figures 3 and 5 (lines 294-206; 315-317), as follows:

“While A and F show distributions of these traits (scaled by subtracting the mean from each value and then dividing the result by the standard deviation) for males (black with dashed borders) and females (white with solid borders) [...]”

Similarly, we added the following to the legend of figure 4 (lines 302-304):

“Panels A and C show these relationships (with variables scaled by subtracting the mean from each value and then dividing the result by the standard deviation) separately for males (dashed line) and females (solid line), each subpanel representing a different phenotyping centre and/or mice strain.”

# Reviewer #3:

[IMPORTANT: please see attachment for fully formatted version]

1. Summary of the research and overall impression

This methods-focused article introduces three novel effect size metrics that quantify differences in higher-order moments of data distributions—specifically skewness, kurtosis, and within-group correlation—between two groups, while claiming to account for sampling error. Using a large dataset of mouse traits, the authors demonstrate the potential of these metrics to support more nuanced biological analyses of sex differences, extending beyond the conventional comparison of group means. This application is engaging and relevant, and the manuscript is generally well written and scholarly in tone.

While the underlying statistical concepts are familiar to statisticians and data analysts, the use of standardized effect sizes to quantify differences in skewness, kurtosis, and correlation in a meta-analytic framework is likely to be novel to many readers in the biological sciences. As such, the manuscript may offer value by expanding the toolbox available for quantitative synthesis across studies.

However, I am uncertain whether the proposed metrics address a clearly defined or pressing biological question, as expected by the journal’s scope. Nevertheless, this may be the first attempt to present standardized effect sizes for higher-order distributional properties to a broader audience, along with accessible R code for implementation. I can envision their integration into future meta-analyses involving two-group comparisons.

A major concern relates to the recommended minimum sample size of 50 per group, which is difficult to achieve in many preclinical studies. While I recognize the statistical necessity, this limitation may substantially constrain the applicability of the proposed approach in real-world settings.

Overall, I see potential in the methodological contribution, though I remain uncertain about its future uptake in the biological community.

R3.1: We thank the reviewer for their words. While we agree these approaches will often require large samples (as shown in our newly added simulations, see R1.1) we believe these limitations (which we acknowledge in our paper) will be overcome in the very near future with the advent of raw data deposition and high-throughput phenotyping. As such, we think this is the perfect time to introduce them to a wide readership so that they begin to think about their applicability under a wide-range of new settings.

2. Evidence and examples

Major Issues.

1. Could the authors please clarify what is meant by “accounting for sampling error” as stated in the abstract? In the current version of the manuscript, only the sampling variance of the proposed metrics is reported, which does not by itself constitute a correction for sampling error. If the authors apply any method to reduce or correct for sampling error—such as bias correction, shrinkage estimation, or bootstrapping—this should be clearly described in the methods section. If not, the claim in the abstract should be rephrased to avoid confusion.

R3.2: We apologise for the confusion. We have now removed “while accounting for sampling error” from this sentence (line 44).

2. Could the authors please clarify the origin of formulas (1)–(12)? It is currently unclear whether these equations are derived by the authors, adapted from existing literature, or directly taken from another source. A mathematical reference or derivation would help readers assess the theoretical foundation of the proposed metrics. In the provided R code, reference is made to Pick et al. [18]; if the formulas are based on or adapted from that work, this should be explicitly stated in the manuscript.

R3.3: We have now added references for equations based on previous work, for skewness and kurtosis (eqs. 1-4: Joanes & Gill 1998) and for Fisher’s Zr (eqs. 9-10 Hedges & Olkin 1985). Other equations, related to the effect sizes we are proposing (eqs. 5-8, 11-12), are original.

Joanes DN, Gill CA. Comparing measures of sample skewness and kurtosis. J R Stat Soc Ser D (The Statistician). 1998;47: 183–189. doi:10.1111/1467-9884.00122

Hedges L V., Olkin I. Statistical methods for meta-analysis. Amsterdam, Netherlands: Elsevier; 1985. doi:10.1016/C2009-0-03396-0

3. Would the authors consider citing prior work proposing standardized skewness metrics to enhance comparability? I found for example that Malgady (2007) (How skewed are psychological data? A standardized index of effect size, J Gen Psychol.

2007 Jul;134(3):355–359. doi: 10.3200/GENP.134.3.355-360) introduced a 0–1 scaled index of skewness aimed at improving comparability across studies. Including this reference would acknowledge that using skewness as a comparative metric is not entirely new and place the current work in the context of previous efforts.

R3.4: We thank the reviewer for suggesting this reference. We have now included it as reference [29] in lines 88-91:

“Although location-scale-shape models (e.g., [26–28]) may be used to explore between-group differences (e.g., males and females) in skewness, kurtosis, or within-group correlations, there are no effect size statistics that can easily measure such differences (but see also [29]).”

Minor Issues

• The phrase “The time is particularly ripe for analyses” is too colloquial and should be rephrased to maintain a formal academic tone.

R3.5: We apologise for the colloquial language we used. We replaced this sentence with:

“The present moment is particularly conducive for analyses using these new effect sizes because the individual-level data (e.g., individual participant data [30,31]) required for their calculation are increasingly available from new technological advances that allow faster data collection and sharing (e.g., automated phenotyping).”
