# Supplementary material for: New approaches to meta-analyze differences in skewness, kurtosis, and correlation
Source: PLoS Biol. 2026 Feb 13;24(2):e3003653. doi: 10.1371/journal.pbio.3003653 (PMC12928594; doi:10.1371/journal.pbio.3003653)
Supplement: Attachment — Submitted filename: plos_biology_response_letter_to_reviewers_round_3.docx [file pbio.3003653.s010.docx]

Sydney, 14 January 2026

Dear Dr. Roberts,

Thank you for the opportunity to submit a revised version of our manuscript ‘*Beyond sex differences in the mean: new approaches to meta-analyse differences in skewness, kurtosis, and correlation*’. We thank you and the reviewers for their helpful and insightful suggestions, which have made the manuscript stronger. We note that our extensive supplementary material (<https://pietropollo.github.io/new_effect_size_statistics/>) contains most of the content that addresses comments made by reviewers. Please find our responses (in black) to all comments (in grey) below, with excerpts from the manuscript in blue. Line numbers are from the clean version of the manuscript.

Dear Dr Pollo,

Thank you for your patience while we considered your revised manuscript "Beyond sex differences in the mean: new approaches to meta-analyse differences in skewness, kurtosis, and correlation" for consideration as a Methods and Resources at PLOS Biology. Your revised study has now been evaluated by the PLOS Biology editors, the Academic Editor and the original reviewers.

You'll see that reviewer #1 now signs off enthusiastically with no further requests. Reviewer #2 complains that you failed to respond to some of his previous comments (you might want to check back and respond to these retrospectively), and while he says that the paper is much improved, he pushes for further analyses and more rigorous reporting of your meta-analyses. Reviewer #3 thinks that their concerns have been minimally addressed (again, you should look back and see if you can address these more fully), but the large sample numbers required mean that his/her scepticism about uptake prevents them from giving a firm recommendation.

In light of the reviews, which you will find at the end of this email, we are pleased to offer you the opportunity to address the remaining points from the reviewers in a revision that we anticipate should not take you very long. We will then assess your revised manuscript and your response to the reviewers' comments with our Academic Editor aiming to avoid further rounds of peer-review, although we might need to consult with the reviewers, depending on the nature of the revisions.

In addition to these revisions, you may need to complete some formatting changes, which you will receive in a follow up email. A member of our team will be in touch with a set of requests shortly. If you do not receive a separate email within a few days, please assume that checks have been completed, and no additional changes are required.

We expect to receive your revised manuscript within 1 month. Please email us (plosbiology@plos.org) if you have any questions or concerns, or would like to request an extension.

At this stage, your manuscript remains formally under active consideration at our journal; please notify us by email if you do not intend to submit a revision so that we withdraw the manuscript.

**IMPORTANT - SUBMITTING YOUR REVISION**

Your revisions should address the specific points made by each reviewer. Please submit the following files along with your revised manuscript:

1. A 'Response to Reviewers' file - this should detail your responses to the editorial requests, present a point-by-point response to all of the reviewers' comments, and indicate the changes made to the manuscript.

*NOTE: In your point-by-point response to the reviewers, please provide the full context of each review. Do not selectively quote paragraphs or sentences to reply to. The entire set of reviewer comments should be present in full and each specific point should be responded to individually.

You should also cite any additional relevant literature that has been published since the original submission and mention any additional citations in your response.

2. In addition to a clean copy of the manuscript, please also upload a 'track-changes' version of your manuscript that specifies the edits made. This should be uploaded as a "Revised Article with Changes Highlighted " file type.

*Resubmission Checklist*

When you are ready to resubmit your revised manuscript, please refer to this resubmission checklist: https://plos.io/Biology_Checklist

To submit a revised version of your manuscript, please go to https://www.editorialmanager.com/pbiology/ and log in as an Author. Click the link labelled 'Submissions Needing Revision' where you will find your submission record.

Please make sure to read the following important policies and guidelines while preparing your revision:

*Published Peer Review*

Please note while forming your response, if your article is accepted, you may have the opportunity to make the peer review history publicly available. The record will include editor decision letters (with reviews) and your responses to reviewer comments. If eligible, we will contact you to opt in or out. Please see here for more details:

https://blogs.plos.org/plos/2019/05/plos-journals-now-open-for-published-peer-review/

*PLOS Data Policy*

Please note that as a condition of publication PLOS' data policy (http://journals.plos.org/plosbiology/s/data-availability) requires that you make available all data used to draw the conclusions arrived at in your manuscript. If you have not already done so, you must include any data used in your manuscript either in appropriate repositories, within the body of the manuscript, or as supporting information (N.B. this includes any numerical values that were used to generate graphs, histograms etc.). For an example see here: http://www.plosbiology.org/article/info%3Adoi%2F10.1371%2Fjournal.pbio.1001908#s5

*Blot and Gel Data Policy*

We require the original, uncropped and minimally adjusted images supporting all blot and gel results reported in an article's figures or Supporting Information files. We will require these files before a manuscript can be accepted so please prepare them now, if you have not already uploaded them. Please carefully read our guidelines for how to prepare and upload this data: https://journals.plos.org/plosbiology/s/figures#loc-blot-and-gel-reporting-requirements

*Protocols deposition*

To enhance the reproducibility of your results, we recommend that if applicable you deposit your laboratory protocols in protocols.io, where a protocol can be assigned its own identifier (DOI) such that it can be cited independently in the future. Additionally, PLOS ONE offers an option for publishing peer-reviewed Lab Protocol articles, which describe protocols hosted on protocols.io. Read more information on sharing protocols at https://plos.org/protocols?utm_medium=editorial-email&utm_source=authorletters&utm_campaign=protocols

Thank you again for your submission to our journal. We hope that our editorial process has been constructive thus far, and we welcome your feedback at any time. Please don't hesitate to contact us if you have any questions or comments.

Sincerely,

Roli

Roland Roberts, PhD

Senior Editor

PLOS Biology

rroberts@plos.org

----------------------------------------------------------------

# REVIEWERS' COMMENTS:

## Reviewer #1:

I am happy with the changes and how the authors answered my question. Congratulations to this nice piece of work.

R1.1: We are glad that our responses satisfied the reviewer, and we thank them for such positive feedback.

## Reviewer #2:

[identifies himself as Andrew W Brown]

The manuscript, "Beyond sex differences in the mean: new approaches to meta-analyse differences in skewness, kurtosis, and correlation," made many improvements. Although the manuscript seemed to address some of my concerns, oddly there were multiple paragraphs of my remarks that had no responses at all in the response to reviewers.

R2.1: We apologise for missing points previously raised by the reviewer. We believe that our responses to the comments below now correctly address them. Nonetheless, some comments were already addressed in our extensive supplement (<https://pietropollo.github.io/new_effect_size_statistics/>), which Reviewer 2 may have missed. We have now tried to refer back to this document when applicable.

The manuscript does a much better job assessing the proposed metrics with the simulations. The authors proposed and tested many different situations (1200 scenarios for delta_sk and delta_ku; 768 for delta_Zr), but they do not report whether the metrics are acceptable or particularly problematic, other than based on sample size. Is there any other inference that can be drawn from these different scenarios? As an analogy, consider the different recommendations for assessing differences in central tendency whether data are or are not normally distributed, groups are or are not homoscedastic, sample sizes are or are not equal, etc…

R2.2: Δ*Zr* and Δ*sk* perform extremely well across nearly all scenarios with little variation to explore, as shown in our simulations (Fig. 1; Fig. S1). In contrast, *Δku* (or excess kurtosis to be more precise) shows more variability across simulated scenarios.

As suggested by Reviewer 2, we have attempted to explore when *Δku* performs poorly, and identify possible reasons for this. Bias is not related to mean or variance differences between groups (see figure below, and Fig. S2, Fig. S3). We also explored Δ*sk,* but as indicated above, there was less variation and the results were similar so we present only *Δku* here.

The variation we see in *Δku* is likely related to challenges in estimating kurtosis, which have been described elsewhere (Wright & Herrington 2011). Given the substantial simulations we have done already, along with space limitations, we have made clear that exploring *Δku* should be a priority for future work (lines lines 357-358). However, our simulations and recommendations show that it can work when *Δku* is < 1. Having said this, our simulation shows the point estimate is unbiased, so we will just need to use sample size based weights rather than the standard inverse-variance meta-analysis. Therefore, we recommend using sample-size-based meta-analysis for *Δku*, adjusting our examples to reflect this change (lines 237-240):

“Given the challenges in estimating Δ*ku*, and the poor properties of its sampling variance [42], we recommend weighted meta-analytic models using sample size instead of sampling variance (see supplementary material and [41]).”

We also added the following information in lines 198-201:

“Note that our simulations did not explore differences in sample size between groups. However, many groups being compared in meta-analyses have the same or very similar sample size. Additionally, simulations often show relatively small impacts of unbalanced sample sizes [40,41], which is why we originally did not vary sample size between groups in our simulations.”

The authors estimate 95% CIs for their metrics in their meta-analysis, but, as I mentioned before, the authors have not demonstrated or established (e.g., through citing other work) that their approach results in appropriate interval coverage. They mention bias and relative bias, which is good to see, but they have not demonstrated or made the case that these extend to what is needed for within-study assessment for any confidence interval that would be included in a meta-analysis.

R2.3: We agree and thank the reviewer for catching this oversight. We have now added coverage plots from our simulations to the main manuscript and supplement (see Figure 2).

The meta-analysis itself, then, serves as a demonstration of the use of the metrics in practice, but still does not assess the performance of the individual study metrics in a meta-analytic context. The raw data are there, so comparing the meta-analyzed effect estimate against an individual-rodent-data meta-analysis would inform how well the meta-analyzed metrics match the real data. Additional simulations could inform the performance, as well: How does a meta-analysis perform under the null? How does it perform under different known effect sizes, different variances, and sample sizes? How does it perform under heterogeneity of effects? As I mentioned before, this could be assessed through simulations based on the raw, individual-rodent data by creating subsamples, changing the effect size, variance, and sample size, and introducing heterogeneity of effect sizes.

R2.4: Thank you for this suggestion. We originally skipped formal validation of the meta-analysis itself as it is we thought it was likely to be beyond the scope of our original manuscript. However, we have now updated the supplementary materials with an additional section “Meta-analysis validation” that uses simulated subsamplings of the original data (with varying known effect size in skewness/kurtosis difference). We assess the estimation bias of meta-analysis for both effect size measures, as well as Type II error of inference (overall realised power).

We also compared the summary-level meta-analysis (our new effect sizes) with IPD-style analysis (where individual scores of “skewness” and “kurtosis” were computed as the 3rd/4th powers of the standardised phenotype).

Overall, our assessment demonstrates that:

(i) summary-level meta-analysis agrees with IPD meta-analysis for skewness, but much less so for kurtosis;

(ii) the bias of estimating the average effect-size is negligible for skewness difference, and remains substantial for kurtosis difference (independently of the meta-analysis sample size);

(iii) power for estimation of skewness differences quickly plateaus above 80% for meta-analyses with >40 studies, assuming moderate to large effect size magnitudes, whereas for kurtosis power seems to be overestimated due to distributional issues we discuss in, e.g., R2.2. Artificially introduced heterogeneity has no effect on bias in delta-skewness estimation, but as it increases it tends to reduce the bias in delta-kurtosis estimation.

For more details - see Figs. S24 - S27 in the Supplementary Online Materials.

Even without assessing the performance of these metrics in meta-analysis, the meta-analysis in Figure 3 and the text describing the analyses are missing most of the information that would be expected of a forest plot of a random effects meta-analysis: sample size, effect estimates, heterogeneity estimates and tests, etc… The reporting of the meta-analyses is unsatisfactory by norms and standards (e.g., PRISMA). For instance, in line 259 the authors call out a specific phenotyping centre, but never report whether there was significant heterogeneity to justify evaluating individual effect estimates. In 259-261, the manuscript reports greater skewness in fat mass and heart weight, then follow with the fact that the values overlap zero. They therefore have not specified what their criteria are for concluding differences, but even if they had the meta-analyzed estimates are close to null with wide confidence intervals, making it strange to make conclusions about differences in skewness. The article's title purports to present new approaches, and thus it is incumbent on the authors to demonstrate how to use and rigorously report a meta-analysis of these metrics.

R2.5: We apologise for the lack of details related to the meta-analyses conducted in our study, and for their faulty interpretation. We have now provided heterogeneity estimates for all meta-analytical models fitted in our study in Table 1, showing that heterogeneity was substantial and largely explained by phenotyping centre and/or mice strain in several cases. We also added sample sizes and mean point estimates to Figures 3, 4, and 5. Lastly, we corrected our interpretations of mean effect sizes related to skewness and kurtosis, as we agree that when estimates overlap zero they should be interpreted as non-different from zero.

Minor point: Lines 69-71 are presumptive. We do not know why people focus on means, but 'obsession' seems an overstatement, and it is more likely that means are just what people were taught.

R2.6: We apologise for such forceful language. We have now modified this sentence as follows (line 69-71):
“A fixation on mean differences has been present for a long time in science because researchers tend to focus on dimorphism in trait averages (e.g., [11]), lack sufficiently powerful data, or have limited statistical tools available (or difficulty to use them).”

Line 245: it is unclear why data were limited. For meta-analysis purposes, it seems it would make sense to include independent samples with varying sizes, which would represent realistic literature.

R2.7: The samples we used for our meta-analytical examples in this study (i.e. mice from different phenotyping centers and strains) do vary in sample size. We do not quite understand what Reviewer 2 meant to suggest, as the line mentioned by them is:

“The IMPC dataset contains data from multiple phenotyping centres and mice strains, so we selected the ones with the most data points for our analyses here, computing the aforementioned effect size statistics separately for each one of them.”

I invited a junior colleague to provide some thoughts, and he remarked:

They used jackknife estimator to estimate bias under finite sample size and normality assumption is violated, which is more common in real life dataset. And I know it's straight forward to prove that the proposed difference of sample skewness and kurtosis are unbiased under the normality assumption, so perhaps a brief comment on this point would be helpful (I might have missed it if it's mentioned elsewhere).

R2.8: Apologies, but we are not sure we completely understand exactly what Reviewer 2 is suggesting here. Maybe this comment is referring to the fact that resampling violates normality assumptions, which is indeed the case. We have now found a paper (Wright & Herrington 2011) showing that, for skewness and kurtosis, the general SE formula does not work as they assume normality and their findings do match our findings. We mention this in lines 231-232.

We have made clearer recommendations about how to best do meta-analysis of our effect sizes given that the sampling distribution of some of them are skewed (lines 237-240).

Wright, D.B., Herrington, J.A. Problematic standard errors and confidence intervals for skewness and kurtosis. *Behav Res* **43**, 8–17 (2011). <https://doi.org/10.3758/s13428-010-0044-x>

This paper might be focusing on the practical side of this method (finite sample), but I am interested to see if these estimators are consistent (i.e. as sample size goes to infinity, the difference in sample skewness converge in probability to the difference in population skewness), which might be beyond the scope of this paper.

R2.9: We have explored how our effect size statistics perform across a fairly large range of sample sizes in our simulations (n = 10 – 500 / group), and provide this in the manuscript with more details in the supplement.

## Reviewer #3:

My review points have all been addressed, though on a very short note within the manuscript (like citing the reference [29] on a standardized skewness metrics, without any elaboration). Overall, I see potential in the methodological contribution, though I remain uncertain about its future uptake in the biological community. Required sample sizes are huge, in the field of preclinical research at least. That is why I stay undecided and can unfortunately neither give a clear recommendation nor recommend rejecting the manuscript.

R3.1: We appreciate the reviewer’s feedback. We note that the large sample sizes required to calculate the effect sizes we propose can indeed limit their application in some fields. However, such sample sizes are increasingly available (the era of Big Data is already here) and also more commonly achieved in ecology and evolution (our own field). Thus, while we understand the reviewer’s reservations, we maintain that our study offers valuable tools for the scientific community. Also, what Reviewer 3 says is exactly the reason why people have not done it. Yet, as we argue in our manuscript, the time is ripe and it is an excellent timing to empower researchers with the method we are proposing.
