# Supplementary material for: New approaches to meta-analyze differences in skewness, kurtosis, and correlation
Source: PLoS Biol. 2026 Feb 13;24(2):e3003653. doi: 10.1371/journal.pbio.3003653 (PMC12928594; doi:10.1371/journal.pbio.3003653)
Supplement: Attachment — Submitted filename: plos_biology_response_letter_to_reviewers_round_4.docx [file pbio.3003653.s011.docx]

Sydney, 27 January 2026

Dear Dr. Roberts,

Thank you for accepting the revised version of our manuscript ‘*New approaches to meta-analyse between-group differences in skewness, kurtosis, and correlation*’ for publication as a Methods and Resources Article in *PLOS Biology*. Please find our responses (in black) to all your comments (in grey) below, with excerpts from the manuscript in blue. Line numbers are from the clean version of the manuscript.

Dear Dr Pollo,

Thank you for your patience while we considered your revised manuscript "Beyond sex differences in the mean: new approaches to meta-analyse differences in skewness, kurtosis, and correlation" for publication as a Methods and Resources at PLOS Biology. This revised version of your manuscript has been evaluated by the PLOS Biology editors and the Academic Editor.

Based on our Academic Editor's assessment of your revision, we are likely to accept this manuscript for publication, provided you satisfactorily address the following data and other policy-related requests.

IMPORTANT - please attend to the following:

a) We need the Title to avoid the colon and to contain an active verb. We suggest: "New approaches to meta-analyse differences in skewness, kurtosis and correlation reveal sex differences beyond the mean" or (presumably it's generalisable beyond sex differences?): "New approaches to meta-analyse differences in skewness, kurtosis and correlation reveal between-group differences beyond the mean"

R1: We have now changed our title to “New approaches to meta-analyse differences in skewness, kurtosis and correlation” (line 1).

b) Many thanks for supplying the data and code needed to recreate the Figures in Github. However, because Github depositions can be readily changed or deleted, please make a permanent DOI’d copy (e.g. in Zenodo) and provide this URL (see below).

R2: We have now deposited the supplementary material in Zenodo (https://zenodo.org/records/18386956), as requested.

c) Please cite the location of the data clearly in all relevant main and supplementary Figure legends, e.g. “The data and code needed to generate this Figure can be found in https://zenodo.org/records/XXXXXXXX”

R3: We have now inserted the suggested statement along with the link to the supplementary material in Zenodo in all figure legends of the manuscript.

d) I note that you mention me and the reviewers (“Andrew W. Brown, two anonymous referees, and the editor Roland Roberts”) in the Acknowledgements. While we appreciate the sentiment, this is against PLOS policy, so please could you remove this?

R4: Apologies, we have now completely removed the sentence mentioned.

e) Please include the URLs of your funders in the Financial Disclosure statement.

R5: We have now included the URLs of our funders in the Financial Disclosure statement (lines 409-416).

As you address these items, please take this last chance to review your reference list to ensure that it is complete and correct. If you have cited papers that have been retracted, please include the rationale for doing so in the manuscript text, or remove these references and replace them with relevant current references. Any changes to the reference list should be mentioned in the cover letter that accompanies your revised manuscript.

R6: We have updated reference #34 (lines 537-539), as it was a preprint that has now been published.

In addition to these revisions, you may need to complete some formatting changes, which you will receive in a follow up email. A member of our team will be in touch with a set of requests shortly. If you do not receive a separate email within a few days, please assume that checks have been completed, and no additional changes are required.

We expect to receive your revised manuscript within two weeks.

To submit your revision, please go to <https://www.editorialmanager.com/pbiology/> and log in as an Author. Click the link labelled 'Submissions Needing Revision' to find your submission record.

Your revised submission must include the following:

-  a cover letter that should detail your responses to any editorial requests, if applicable, and whether changes have been made to the reference list

-  a Response to Reviewers file that provides a detailed response to the reviewers' comments (if applicable, if not applicable please do not delete your existing 'Response to Reviewers' file.)
-  a track-changes file indicating any changes that you have made to the manuscript.

NOTE: If Supporting Information files are included with your article, note that these are not copyedited and will be published as they are submitted. Please ensure that these files are legible and of high quality (at least 300 dpi) in an easily accessible file format. For this reason, please be aware that any references listed in an SI file will not be indexed. For more information, see our Supporting Information guidelines: <https://journals.plos.org/plosbiology/s/supporting-information>

*Published Peer Review History*

Please note that you may have the opportunity to make the peer review history publicly available. The record will include editor decision letters (with reviews) and your responses to reviewer comments. If eligible, we will contact you to opt in or out. Please see here for more details:
<https://plos.org/published-peer-review-history/>

*Press*
Should you, your institution's press office or the journal office choose to press release your paper, please ensure you have opted out of Early Article Posting on the submission form. We ask that you notify us as soon as possible if you or your institution is planning to press release the article.

*Protocols deposition*

To enhance the reproducibility of your results, we recommend that if applicable you deposit your laboratory protocols in protocols.io, where a protocol can be assigned its own identifier (DOI) such that it can be cited independently in the future. Additionally, PLOS ONE offers an option for publishing peer-reviewed Lab Protocol articles, which describe protocols hosted on protocols.io. Read more information on sharing protocols at <https://plos.org/protocols?utm_medium=editorial-email&utm_source=authorletters&utm_campaign=protocols>

Please do not hesitate to contact me should you have any questions.

Sincerely,

Roli Roberts

Roland Roberts, PhD

Senior Editor

rroberts@plos.org

PLOS Biology
