# Supplementary material for: New approaches to meta-analyze differences in skewness, kurtosis, and correlation
Source: PLoS Biol. 2026 Feb 13;24(2):e3003653. doi: 10.1371/journal.pbio.3003653 (PMC12928594; doi:10.1371/journal.pbio.3003653)
Supplement: Attachment — Submitted filename: Reviewer Comments for PBIOLOGY.pdf [file pbio.3003653.s008.pdf]

Reviewer Comments for Manuscript Number PBIOLOGY-D-25-01173R1
"Beyond sex differences in mean: meta-analysis of differences in skewness, kurtosis, and correlation"

1. Summary of the research and overall impression

This methods-focused article introduces three novel effect size metrics that quantify differences in higher-order moments of data distributions—specifically skewness, kurtosis, and within-group correlation—between two groups, while claiming to account for sampling error. Using a large dataset of mouse traits, the authors demonstrate the potential of these metrics to support more nuanced biological analyses of sex differences, extending beyond the conventional comparison of group means. This application is engaging and relevant, and the manuscript is generally well written and scholarly in tone.

While the underlying statistical concepts are familiar to statisticians and data analysts, the use of standardized effect sizes to quantify differences in skewness, kurtosis, and correlation in a meta-analytic framework is likely to be novel to many readers in the biological sciences. As such, the manuscript may offer value by expanding the toolbox available for quantitative synthesis across studies.

However, I am uncertain whether the proposed metrics address a clearly defined or pressing biological question, as expected by the journal’s scope. Nevertheless, this may be the first attempt to present standardized effect sizes for higher-order distributional properties to a broader audience, along with accessible R code for implementation. I can envision their integration into future meta-analyses involving two-group comparisons.

A major concern relates to the recommended minimum sample size of 50 per group, which is difficult to achieve in many preclinical studies. While I recognize the statistical necessity, this limitation may substantially constrain the applicability of the proposed approach in real-world settings.

Overall, I see potential in the methodological contribution, though I remain uncertain about its future uptake in the biological community.

2. Evidence and examples

Major Issues.

1. **Could the authors please clarify what is meant by “accounting for sampling error” as stated in the abstract?** In the current version of the manuscript, only the sampling variance of the proposed metrics is reported, which does not by itself constitute a correction for sampling error. If the authors apply any method to reduce or correct for sampling error—such as bias correction, shrinkage estimation, or bootstrapping—this should be clearly described in the methods section. If not, the claim in the abstract should be rephrased to avoid confusion.

2. **Could the authors please clarify the origin of formulas (1)–(12)?** It is currently unclear whether these equations are derived by the authors, adapted from existing literature, or directly taken from another source. A mathematical reference or derivation would help readers assess the theoretical foundation of the proposed metrics. In the provided R code, reference is made to Pick et al. [18]; if the formulas are based on or adapted from that work, this should be explicitly stated in the manuscript.

3. **Would the authors consider citing prior work proposing standardized skewness metrics to enhance comparability?** I found for example that Malgady (2007) (*How skewed are psychological data? A standardized index of effect size, J Gen Psychol.* 2007 Jul;134(3):355–359. doi: 10.3200/GENP.134.3.355-360) introduced a 0–1 scaled index of skewness aimed at improving comparability across studies. Including this reference would acknowledge that using skewness as a comparative metric is not entirely new and place the current work in the context of previous efforts.

Minor Issues

- The phrase “The time is particularly ripe for analyses” is too colloquial and should be rephrased to maintain a formal academic tone.
